# Immunosuppressive Microenvironment Reprogramming by Synergistic Sonodynamic Therapy of Phthalocyanine‐MOF Hybrids for Hepatocellular Carcinoma

**DOI:** 10.1002/EXP.20250074

**Published:** 2026-02-22

**Authors:** Han Wu, Lihui Gu, Jiahao Xu, Chuyue Zhang, Mingda Wang, Chao Li, Lanqing Yao, Yongkang Diao, Yuchen Li, Fujie Chen, Huixuan Fan, Yuze Zhao, Feng Shen, Tian Yang

**Affiliations:** ^1^ Department of Hepatobiliary Surgery Eastern Hepatobiliary Surgery Hospital Naval Medical University Shanghai China; ^2^ Clinical Research Institute Eastern Hepatobiliary Surgery Hospital Naval Medical University Shanghai China; ^3^ National University Hospital National University of Singapore Singapore Singapore; ^4^ The First Affiliated Hospital of Harbin Medical University Harbin Medical University Harbin China

**Keywords:** hepatocellular carcinoma, metal‐organic frame sensitizer, reactive oxygen species, single‐cell immune repertoire, sonodynamic therapy

## Abstract

Hepatocellular carcinoma (HCC) remains a critical global health challenge with limited treatment efficacy hindered by therapy resistance and hypoxia‐induced immune suppression. Tumor heterogeneity and low immune cell reactivity often lead to tolerance and failure of immunotherapy. Sonodynamic therapy (SDT) has emerged as a promising precision treatment with non‐invasive characteristics and localized tumor targeting, offering potential for remodeling the immunosuppressive tumor microenvironment. This study introduces a novel phthalocyanine‐metal‐organic framework hybrid (Pc@Zr‐MOF) designed for SDT and immune regulation in HCC. By enhancing the efficiency of ultrasonic conversion and synergy, Pc@Zr‐MOF induces robust tumor cell apoptosis while simultaneously reshaping the immune landscape. Single‐cell RNA sequencing reveals its ability to promote M1 macrophage polarization and increase cytotoxic T cell infiltration with tumor‐associated macrophages. These effects highlight its dual mechanism of direct tumor eradication and immune microenvironment remodeling. Moreover, Pc@Zr‐MOF demonstrates admirable biocompatibility and minimal off‐target toxicity, which underscores its clinical translational potential. By synergizing SDT with immune reprogramming, this approach addresses hypoxia‐driven resistance and establishes durable anti‐tumor immunity. This study aims to change the current situation of advanced HCC treatment characterized by reconfiguration of tumor cell subpopulations and immunosuppression, thereby bringing a new paradigm to the precise treatment of HCC.

## Graphical abstract

Hepatocellular carcinoma presents a formidable clinical challenge, constructing a hypoxic and immunosuppressive microenvironment that renders conventional therapies largely ineffective. In response, we have developed Pc@Zr‐MOF, a phthalocyanine‐integrated metal‐organic framework hybrid engineered to penetrate this defensive barrier. Upon ultrasound activation, this nanoplatform executes a coordinated dual action: it first generates potent sonodynamic effects that directly induce tumor cell apoptosis, then proceeds to fundamentally reprogram the immune landscape. Single‐cell transcriptomic analysis reveals that Pc@Zr‐MOF drives M1 polarization of tumor‐associated macrophages and facilitates robust infiltration of cytotoxic T lymphocytes, effectively converting an immunosuppressive fortress into an immunologically active territory. This immune remodeling, combined with direct tumor eradication, overcomes hypoxia‐driven resistance and establishes durable antitumor immunity. With demonstrated biocompatibility and minimal off‐target effects, Pc@Zr‐MOF offers a rationally designed strategy to redefine precision treatment for advanced hepatocellular carcinoma.

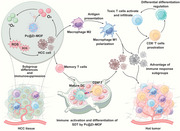

## 1 Introduction

Hepatocellular carcinoma (HCC) is one of the most prevalent and lethal malignancies worldwide [1, 2]. Most patients diagnosed with HCC have already lost the opportunity for surgery, and there are limited therapeutic options for unresectable HCC [3, 4, 5]. Existing local treatment methods, including radiofrequency ablation, transcatheter arterial chemoembolization (TACE), and stereotactic radiotherapy, can effectively inhibit partial unresectable HCC, but their damage to normal liver tissue and inexact efficacy are unsatisfactory [6, 7, 8]. In recent years, rapid developments have been achieved in the discovery of novel targeting drugs and immune system modulating therapies for advanced HCC [9, 10, 11, 12]. Due to the strong tumor heterogeneity of HCC, they often manifest as significant differences in therapeutic effects. Recent studies have shown that HCC cells often exhibit heterogeneity as high and low responses to immunotherapy, mainly due to the differences in self‐differentiation and gene expression of tumor cells [13, 14, 15]. Therefore, enhancing the sensitivity of tumor tissues to immunotherapy and improving local immune suppression are urgent issues that need to be addressed to improve the efficacy of HCC.

Sonodynamic therapy (SDT) has emerged as a promising precision therapy for local cancer treatment leveraging the generation of reactive oxygen species (ROS) induced by sonosensitizers under high‐intensity focused ultrasound [16, 17, 18]. Because ultrasound therapy itself is non‐invasive and non‐injurious to normal tissue, it can effectively reduce the complications of local treatment while ensuring the deep curative effect. However, the clinical application of SDT is still hindered by several challenges, which include the lack of effective water‐soluble sonosensitizers and low efficiency of ultrasonic energy conversion to produce abundant ROS [19, 20]. At the same time, whether locally produced ROS by SDT can reverse treatment tolerance due to the HCC hypoxic microenvironment and modify the tumor cell “don't eat me” signal by regulating cell differentiation and infiltration remains unknown [21, 22, 23]. Therefore, there is a strong driving force to design novel SDT systems to solve these problems while maximizing the precise and sustainable treatment efficacy.

The metal‐organic frameworks (MOFs) with multifunctional high porosity and loading capacity have emerged as a significant sonosensitizer carrier in the cancer SDT field [24, 25, 26]. Since MOFs are designed with a metal catalytic core site and porous structure, such a flexible structure offers controlled release ability and enhanced ROS generation to improve therapeutic effects [27, 28]. However, the use of MOF as an SDT carrier is often limited due to its poor water solubility and dispersion and biological toxicity [29, 30]. In addition, phthalocyanines are considered to be good photosensitizers and sound sensitizers due to their porphyrin ring structure [31, 32, 33]. Due to its poor biocompatibility and light stability, it has not been widely used in ROS‐related therapies such as sonodynamic therapy. On this basis, when applying MOF as the HCC SDT medium with phthalocyanine compounds, it is particularly important to match the suitable pair of metal catalytic cores with good water solubility and dispersion. In addition, in order to effectively reverse therapeutic tolerance caused by hypoxic microenvironments, amplified ROS production is more critical for the long‐term immune memory and immune response after SDT. Enhanced SDT holds promise for reprogramming the tumor microenvironment through antigen presentation and long‐acting anti‐tumor immune activation, thereby enhancing the efficacy of immunotherapy.

**SCHEME 1:**
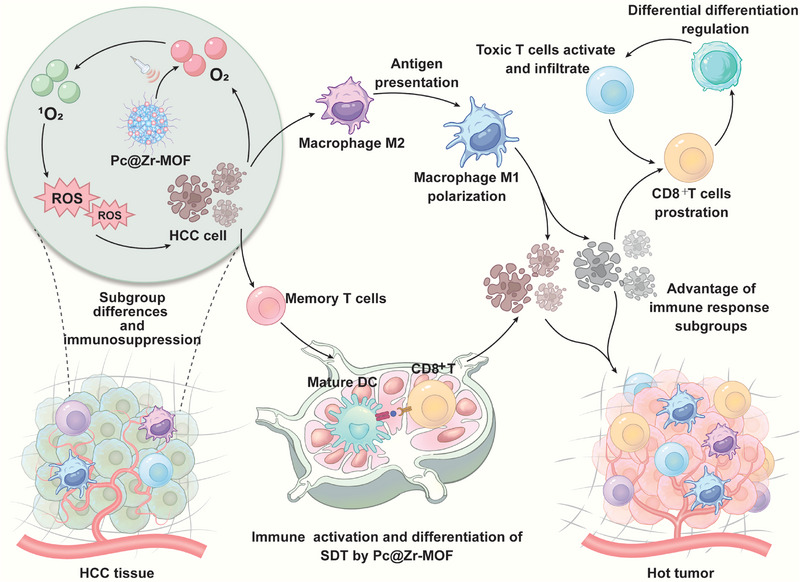
Application and mechanism of ultrasound‐enhanced sonodynamic Pc@Zr‐MOF in local treatment of HCC. The schematic diagram mainly contains the process of ROS treatment in HCC tissues by Pc@Zr‐MOF; SDT regulates immune microenvironment reprogramming and the generation and memory of long‐term anti‐tumor immunity in the body. Immunosuppressive tumors gradually transform into hot tumors under the influence of antigen presentation, changes in the differentiation trend of immune cells, and the long‐term anti‐tumor immune response of the body.

In this study, a synergistic sonodynamic integrating Zr‐MOF with zinc phthalocyanine as a novel sonosensitizer (Pc@Zr‐MOF) was firstly designed for SDT to treat HCC, and sufficient ROS reversed the hypoxic tolerance and immunosuppressive status of HCC by improving tumor differentiation and immune cell infiltration (Scheme 1). The stronger conversion of sound energy of Zr‐MOF and new phthalocyanine catalyzes oxygen‐containing substrate to produce more cytotoxic singlet oxygen (^1^O_2_), which achieves the purpose of SDT therapy while completely improving the local oxygen‐depleted microenvironment and reversing treatment tolerance. Our work attempts to solve the following problems but is not limited to: the synthesis and characterization of the Pc@Zr‐MOF hybrid, evaluation of its sonodynamic and cytotoxic efficacy in preclinical models, and elucidation of the mechanisms underlying the immune modulating effects. By analyzing and comparing the single‐cell sequencing immune repertoire, we aimed to reveal the complex interaction between SDT and the tumor immune microenvironment, paving the way for the rational design of combination therapies that maximize the antitumor immune response. Generally, this work represents a significant step forward in the development of enhanced cancer SDT and the understanding of tumor immune response to biological ROS, which may provide a new paradigm for the accurate, effective, and safe treatment of unresectable HCC.

## 2 Results

### 2.1 Identification of Immunosuppressive Subgroups in HCC and Synthesis of Pc@Zr‐MOF

**FIGURE 1:**
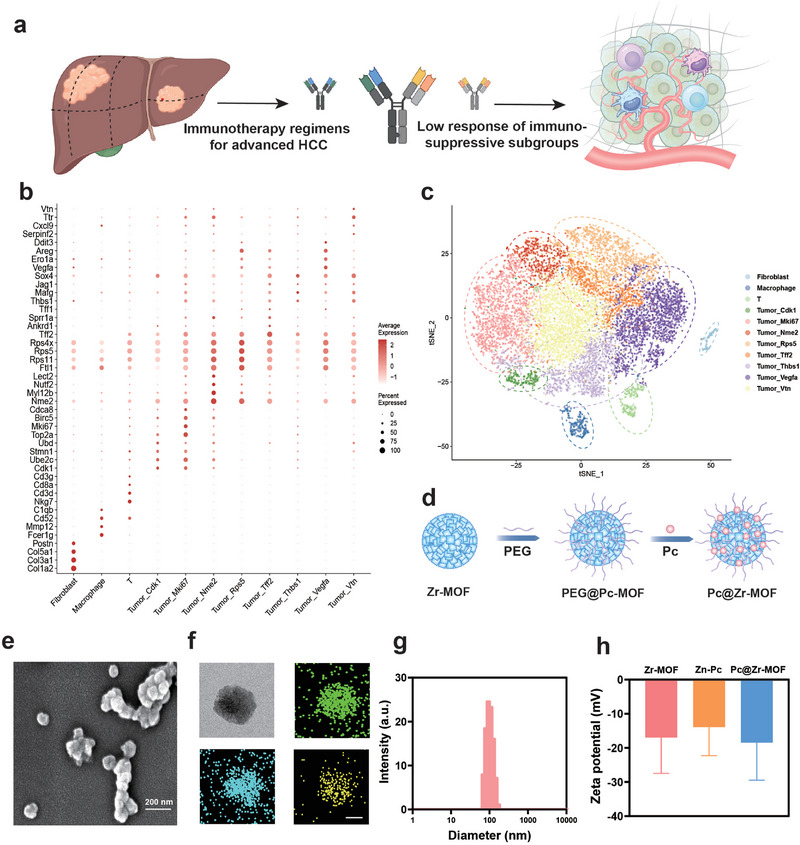
Identification of immunosuppressive subgroups in HCC and Synthesis of Pc@Zr‐MOF. (a) Schematic diagram of the causes of tolerance to tumor immunotherapy in HCC and the tendency of tumor cell differentiation, (b, c) The results of ScRNA mixed analysis on PD‐L1 and PBS treated mice were used to identify tumor cell subsets, (d) Pc@Zr‐MOF Schematic diagram of the design synthesis including PEG modification and Pc loading, (e, f) Pc@Zr‐MOF scanning and transmission electron microscopy images, including multi‐particle and single‐particle images and bright and dark field comparisons with the element planar scanning image of Pc@Zr‐MOF mainly includes Zr, Zn, and O elements (Scale: 200 nm and 50 nm, respectively), (g) The hydrated particle size of Pc@Zr‐MOF is measured by means of DLS, and (h) The zeta potential of Pc@Zr‐MOF and its derivatives. All data are shown as mean value ± SD. (*n* = 3 independent tests).

The efficacy of immunotherapy for HCC varies greatly, which is often due to the large heterogeneity among tumor cells (Figure 1a). Through single‐cell sequencing of tumors with low responsiveness after immunotherapy, it was found that tumor cells could be roughly divided into eight different subgroups and manifested as different expressions of surface markers (Figures 1b,c). In order to remodel the tumor cell clustering and tumor microenvironment, it is necessary to design effective strategies to reduce the proportion of immunosuppressive cell subsets among them. As previously reported [34], Zr‐MOF with good water solubility was synthesized, and PEG was used as a surface modification to ensure the stability of the solution, and then Zn‐Pc was loaded into the pores of Zr‐MOF as a sound‐sensitive enhancer for subsequent treatment (Figure 1d). Due to PEG modification and the good water solubility of Zn‐Pc, the system can be uniformly dispersed in aqueous solution. ICP‐OES is used to test the content of Zn‐Pc in the load and calculate the loading ratio of 34%. Compared with other porphyrin‐based photothermal agents, Zn‐Pc has higher water solubility and loading capacity. Moreover, due to its extended π‐conjugation and stable triplet state, it may have a higher efficiency in converting acoustic energy. After the synthesis of Pc@Zr‐MOF, the enhanced sonodynamic therapy system and its derivatives were characterized by scanning electron microscopy (SEM) and transmission electron microscopy (TEM) (Figure 1e and Supplementary Figures S1a, b). The element distribution of Pc@Zr‐MOF was determined by TEM energy spectrum and element scanning, and the load distribution and quantitative analysis of Zn‐Pc on the surface of MOF was further confirmed (Figure 1f and Supplementary Figure S2). The scaled synthesis of Pc@Zr‐MOF demonstrated excellent batch‐to‐batch reproducibility, a critical advantage over conventional sonosensitizers that often suffer from compositional heterogeneity. This manufacturing consistency enhances the clinical translation potential of our platform. By dynamic light scattering (DLS), it can be found that the average hydrodynamic size of Pc@Zr‐MOF is about 100 nm, and the particle size distribution is relatively concentrated, and the zeta potential of Pc@Zr‐MOF and its related derivatives shows the change of solution potential synthesized step by step (Figures 1g, h).

**FIGURE 2:**
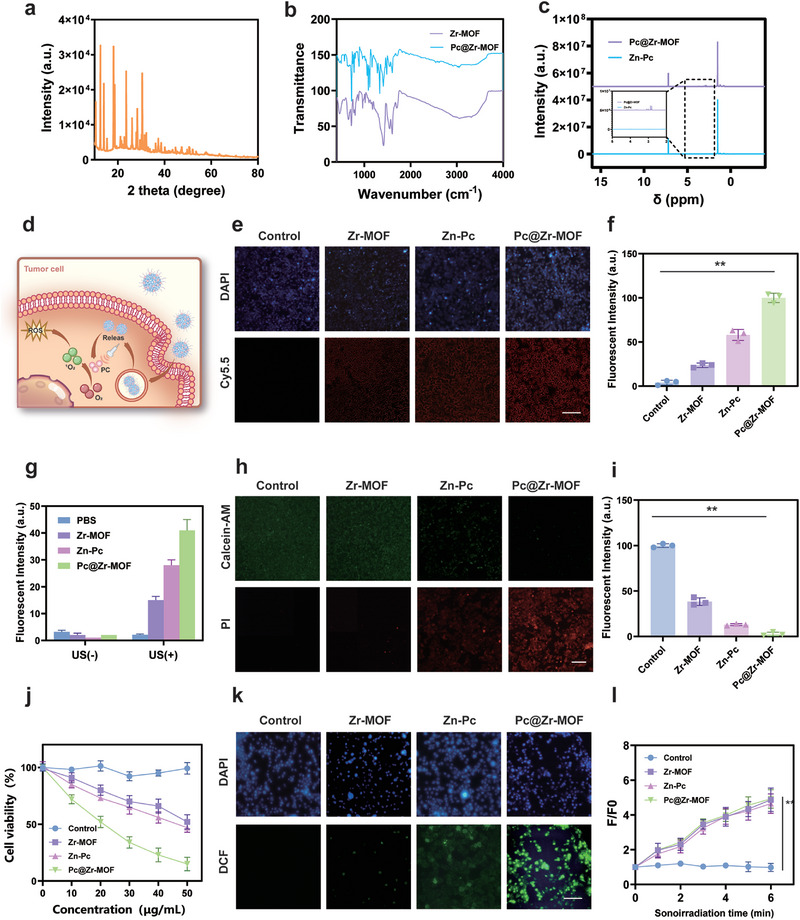
In vitro sonodynamic catalytic validation and phagocytosis and cytotoxicity test of Pc@Zr‐HOF. (a–c) XRD spectrum, Fourier infrared spectra, and hydrogen magnetic resonance spectra with the coordinates of the main outlet locations marked of Pc@Zr‐MOF and its derivatives, (d) Schematic diagram of Pc@Zr‐MOF intracellular actions, including endocytosis, release, and catalytic reactions under sonodynamics, (e, f) Cytophagocytosis assay and corresponding fluorescence quantification of Pc@Zr‐MOF and its derivatives. The related particles were prestained with Cy5.5, and the nuclei were stained with DAPI, (g, h) Fluorescence images of live and dead cells and corresponding fluorescence quantification in different cell groups, (j) CCK‐8 cytotoxicity tests of Pc@Zr‐MOF and its derivatives, and (i, k, l) Fluorescence images and quantification of intracellular ROS production, and time‐dependent curves. All data are shown as mean value ± SD (*n* = 3 independent tests, **p < 0.01).

XRD results also show characteristic peaks different from Zr‐MOF itself, indicating the diversity of its composition (Figure 2a). After the addition of Zn‐Pc, a new characteristic peak appeared at 1485cm^−1^ in Fourier infrared spectroscopy, which belonged to the C = C skeleton stretching vibration peak on the benzene ring, and the new characteristic peaks at 1392 and 1280 cm^−1^ were C = N and C‐N stretching vibration peaks (Figure 2b). The appearance of these characteristic peaks indicates that Zn‐Pc has been successfully loaded on the MOF as expected. We can also verify the successful loading of Zn‐Pc by comparing the characteristic peak of the ^1^H NMR, because a new characteristic peak that does not belong to Pc appears at 2–5 ppm in Pc@Zr‐MOF (Figure 2c). After successfully synthesizing and characterizing Pc@Zr‐MOF, we continued to verify the solution stability and hemolysis tests of the system in order to evaluate the feasibility of its subsequent in vivo application (Supplementary Figures S3a,b). It is not difficult to see from the test results of DLS that the system can maintain long‐term stability in aqueous solution, and the hemolysis test results show that Pc@Zr‐MOF does not cause an obvious hemolytic reaction with the hemolysis rate less than 5%.

We then attempt to use spin polarization density functional theory (DFT) calculations to understand the catalytic reaction of this complex system by calculating the charge states of the excited and ground states (Supplementary Figure S4a). Based on the principle of piezoelectrics, the Pc@Zr‐MOF composite structure generates incessant triggered electron‐hole pair migration and separation under ultrasonic action, which realizes the efficient ^1^O_2_ and hydroxyl radical (•OH) generation (Supplementary Figures S4b,c). Based on DFT calculation, we first studied the feasibility of Pc@Zr‐MOF‐mediated SDT, which laid a foundation for subsequent in vivo and in vitro experiments. In addition, in both cellular and solid tumor models, the efficient production of ROS and its killing effect still depend on the efficient uptake of Pc@Zr‐MOF by HCC cells, as well as the long‐term effects of catalyzed ROS on the tumor immune environment.

### 2.2 Biological Effects and Therapeutic Efficacy for HCC of Pc@Zr‐MOF in Vitro and Vivo

To evaluate the cellular uptake efficiency of the Pc@Zr‐MOF hybrids, fluorescence microscopy was employed to visualize the internalization process in Hepa1‐6 cells. The intracellular endocytosis, release, and catalysis mechanism of Pc@Zr‐MOF is illustrated briefly (Figure 2d). As depicted in Figure 2e, the fluorescence intensity of cells treated with Pc@Zr‐MOF was significantly higher compared to those treated with an aqueous solution of its derivatives. This suggests that the integration of Zn‐Pc into the Zr‐MOF framework not only stabilizes the photosensitizer but also facilitates its delivery into cancer cells due to its surface activity energy changes. The fluorescence intensity quantification confirmed that Pc@Zr‐MOF exhibits the highest cellular uptake among the tested groups (Figure 2f). This enhanced uptake is crucial for the efficacy of SDT, as it ensures sufficient intracellular concentration of the photosensitizer to generate ROS upon ultrasound activation. Upon cellular internalization, the Pc@Zr‐MOF is believed to localize in the cytoplasm, where it can be activated by ultrasound to produce ROS, leading to cellular damage and apoptosis.

To assess the cytotoxicity of Pc@Zr‐MOF‐induced intracellular ROS, the CCK‐8 assay was performed under the therapeutic intensity ultrasound environment (Figure 2j). The results revealed that Pc@Zr‐MOF exhibited minimal cytotoxicity in the absence of ultrasound, indicating high biocompatibility. However, when combined with ultrasound, a significant reduction in cell viability was observed, demonstrating the synergistic effect of the hybrid material and ultrasound in inducing cancer cell death. These findings underscore the potential of Pc@Zr‐MOF as a targeted and efficient SDT agent for HCC. Further evidence of the therapeutic efficacy of Pc@Zr‐MOF was obtained through live/dead cell fluorescent staining (Figures 2g,h). The fluorescence images and corresponding quantifications clearly show a higher proportion of dead cells (red fluorescence) in the Pc@Zr‐MOF group treated with ultrasound compared to the control and other treatment groups. Further concentration gradient and rescue experiments confirmed the production of such concentration‐dependent cytotoxic ROS (Supplementary Figure S5). This result aligns with the CCK‐8 assay, confirming that Pc@Zr‐MOF can effectively induce cancer cell death when activated by ultrasound.

The generation of ROS was evaluated using DCFH‐DA staining in order to visualize the intracellular ROS production (Figures 2i, k). At the same time, in order to test the stability of ROS in the solution, we used an enzyme analyzer to measure the production of ROS under continuous ultrasonic treatment (Supplementary Figure S6). The fluorescence intensity of DCF, revealing the ROS levels, was significantly higher in cells treated with Pc@Zr‐MOF and ultrasound compared to other groups. This suggests that Pc@Zr‐MOF efficiently generates ROS upon ultrasound activation, leading to oxidative stress and subsequent cell death. Figure 2l further quantifies the ROS production over different ultrasound exposure times, demonstrating that ROS generation increases with prolonged ultrasound exposure. This time‐dependent ROS production highlights the importance of optimizing ultrasound parameters to maximize the therapeutic effect of Pc@Zr‐MOF. In summary, in vitro experiments demonstrate that Pc@Zr‐MOF exhibits enhanced cellular uptake, minimal cytotoxicity, and efficient ROS generation upon ultrasound activation. These findings collectively support the potential of Pc@Zr‐MOF as a promising SDT strategy for HCC, which effectively lays the foundation for the subsequent animal experiments.

**FIGURE 3:**
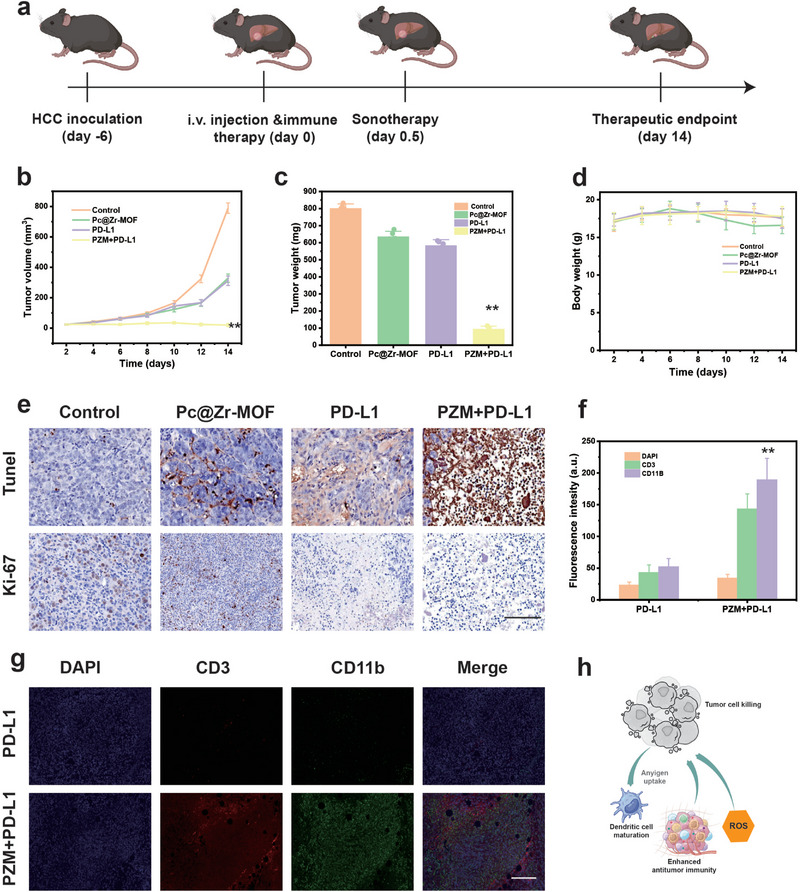
In vivo sonodynamic therapeutic effects of Pc@Zr‐HOF for HCC. (a) Schematic diagram of the treatment process of C57 mouse HCC xenograft tumor model, (b–d) Primary treatment outcomes include tumor volume monitoring, tumor mass measurement, and mouse weight measurement; (e) Immunohistochemical analysis of tumor tissue, including TUNEL and Ki‐67 staining. Scale: 200 µm, (f, g) The immunofluorescence and quantification of tumor tissues after solution sonodynamic therapy with Pc@Zr‐MOF was compared with PBS direct ultrasound therapy. Scale: 200 µm, and (h) Schematic of possible cytological mechanisms of tumor killing and obtaining long‐lasting immunity. All data are shown as mean value ± SD (*n* = 3 independent tests, **p < 0.01).

The in vivo antitumor efficacy of Pc@Zr‐MOF was evaluated in the allograft HCC mouse model, following the experimental timeline illustrated in Figure 3a. Tumor‐bearing mice were intravenously injected with Pc@Zr‐MOF with PD‐L1 (PZM+PD‐L1) or derivative on day 0, followed by SDT 12 h later. Meanwhile, the ultrasound therapy was also simultaneously injected into the abdominal cavity of the mice at day 0.5. Tumor growth was monitored over 14 days to assess sustained therapeutic effects. As shown in Figure 3b and Supplementary Figure S7, mice treated with PZM+PD‐L1 and ultrasound exhibited a significant reduction in tumor volume (< 100 mm^3^) compared to other groups. This was further supported by endpoint tumor weight measurements (Figure 3c), where the PZM+PD‐L1 group displayed the smallest tumor mass (< 0.1 g). These results demonstrate the superior antitumor efficacy of PZM+PD‐L1 when combined with ultrasound, consistent with the in vitro findings. Additionally, body weight measurements revealed no significant weight loss in any treatment group (Figure 3d), indicating that PZM+PD‐L1 is well‐tolerated and does not induce systemic toxicity, a critical factor for clinical translation.

The antitumor effect of PZM+PD‐L1 was further investigated through immunohistochemical analysis (Figure 3e). Tumors from the PZM+PD‐L1 group showed reduced Ki‐67 staining, indicating decreased tumor cell proliferation, and increased infiltration of CD3+ T cells and CD11b+ myeloid cells, suggesting enhanced antitumor immune responses. Finally, immune markers were assessed using fluorescence imaging (Figures 3f, g), revealing significant microenvironment reshaping in tumors treated with PZM+PD‐L1 with SDT compared to controls. These findings collectively demonstrate that SDT of PZM+PD‐L1 not only directly kills tumor cells through ROS generation but also stimulates the immune system to combat residual cancer cells, highlighting its potential as a promising SDT agent for HCC (Figure 3h). It is of great interest to further study the single‐cell transcriptome and immune bank of the tumor microenvironment, and it is more urgent to elucidate the mechanism of long‐term anti‐tumor effect. In fact, the results of the small animals not only demonstrated the feasibility of this approach but also inspired further research on its transformation and application, which requires more consideration of individualized treatment parameters for ultrasound. The successful combination of ROS therapy and immunotherapy is inseparable from the use of appropriate and reasonable ultrasound treatment methods.

### 2.3 Analysis of Single Cell Atlas for Pc@Zr‐MOF Treatment of HCC

**FIGURE 4:**
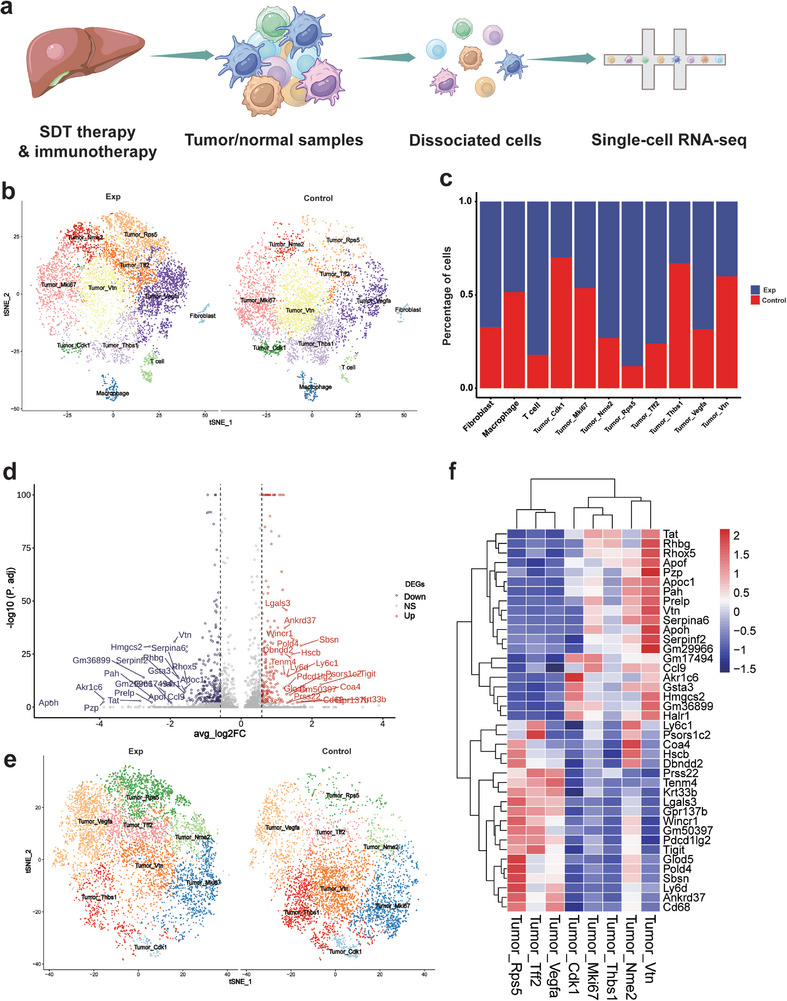
Single‐cell atlas analysis of Pc@Zr‐MOF treatment in HCC. (a) Schematic overview of the experimental single‐cell RNA sequencing (scRNA‐seq) workflow, (b) t‐SNE plot showing the clustering of single cells from HCC samples, (c) Bar plot illustrating the proportional changes in major cell subpopulations (e.g., cytotoxic T cells, NK cells, Tregs, and TAMs) following Pc@Zr‐MOF treatment, demonstrating a shift toward an immunostimulatory tumor microenvironment, (d) Volcano plot highlighting differentially expressed genes between Pc@Zr‐MOF‐treated and control groups (e.g., *TGFB1*, *VEGFA*), (e) The subgroup classification and clustering comparison of tumor cells only included eight different subgroups of HCC cells, and (f) Heatmap displaying the expression of key marker genes across identified cell types, including tumor cells, T cells, macrophages, and stromal cells.

To elucidate the cellular and molecular mechanisms underlying the therapeutic efficacy of Pc@Zr‐MOF in HCC, we performed single‐cell RNA sequencing (scRNA‐seq) on treated tumor (Figure 4a, Supplementary Figure S8). The tumor tissue cell populations treated with enhanced SDT showed significant subgroup differences compared with the control group treated with immunotherapy alone (Figures 4b,c). Differential gene expression analysis between Pc@Zr‐MOF‐treated and control groups highlighted significant downregulation of proliferation‐related genes (MKI67, TOP2A) and upregulation of pro‐apoptotic genes (CASP3, BAX) in tumor cells, indicating that Pc@Zr‐MOF effectively suppresses tumor growth and induces apoptosis (Figure 4d). This indicates that in addition to the changes in microenvironment cell clustering, SDT can also cause alterations in the expression of tumor bulk genes.

Volcano plot analysis further demonstrated significant upregulation of immune activation genes (IFNG, CXCL10) and downregulation of immunosuppressive genes (*TGFB1*, *VEGFA*), suggesting that Pc@Zr‐MOF modulates the immune microenvironment to enhance anti‐tumor immunity. The individual identification of tumor cell subsets can more clearly reveal the reduction of tumor immunosuppressive subsets and the marker genes of each subset (Figures 4e, f). Comparative analysis of cell subpopulation proportions revealed an increase in cytotoxic T cells and natural killer (NK) cells, alongside a decrease in regulatory T cells (Tregs) and tumor‐associated macrophages (TAMs), indicating a shift toward a more immunostimulatory tumor microenvironment (Supplementary Figures S9, 10). Gene set enrichment analysis of differential gene supported these findings, showing enrichment of immune‐related pathways (e.g., interferon signaling) and suppression of tumor‐promoting pathways (e.g., angiogenesis). Single‐cell atlas analysis demonstrates that Pc@Zr‐MOF not only directly targets tumor cells but also reshapes the immune microenvironment to promote anti‐tumor immunity. These findings underscore the potential of Pc@Zr‐MOF as a synergistic therapeutic strategy for HCC, combining SDT with immune modulation to achieve enhanced therapeutic outcomes.

### 2.4 Sonodynamic Therapy of HCC Alters the Tumor Immune Microenvironment Through Macrophage Reprogramming and Polarization

**FIGURE 5:**
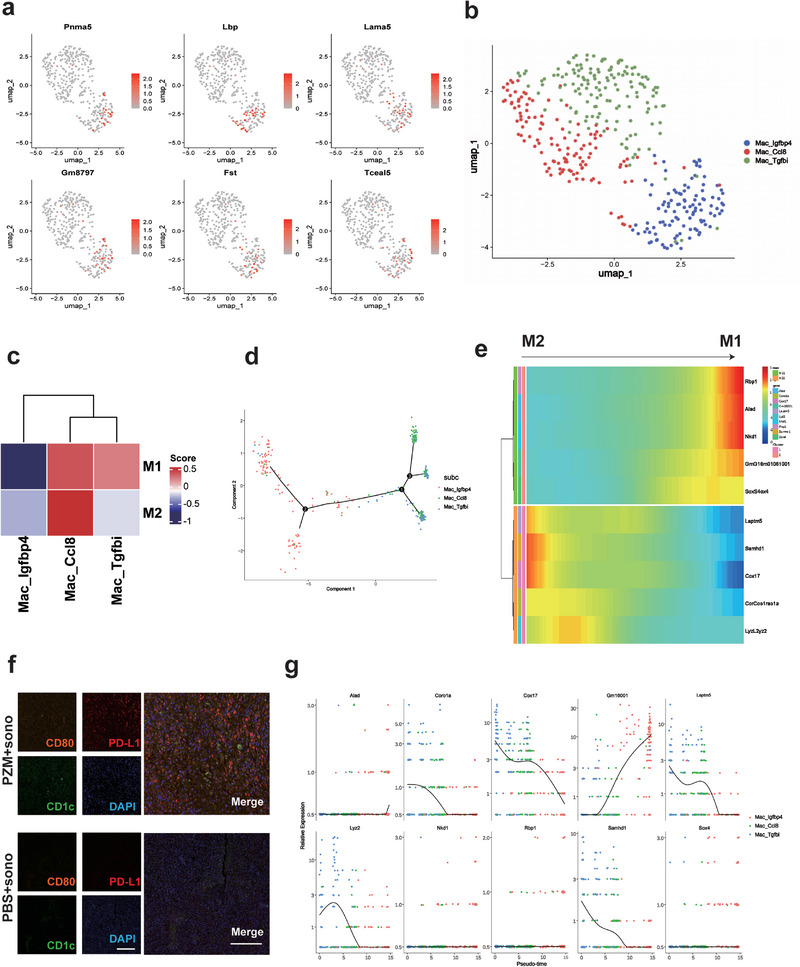
Macrophage reprogramming and polarization analysis of Pc@Zr‐MOF in single‐cell immune repertoire. (a) Distribution of top six differential genes in macrophages on UMAP scatter plot, (b) The UMAP annotation groups of macrophages were mainly divided into *lgfbp4*, *Ccl8* and *Tgfbi* groups, (c) The M1 and M2 polarization typing scores of the three groups of macrophages were performed respectively, (d) A pseudo‐time analysis of the entire population of macrophages reveals its possible developmental direction, (e) Pseudo‐time series analysis of macrophage M1‐M2 polarization reprogramming, (f) Immunofluorescence analysis of antigen presenting cells and T cell activation. Scale: 200 µm, and (g) Pseudotemporal analysis of the top six differential genes in M1 and M2 macrophages.

After obtaining scRNA‐seq and grouping the cells and analyzing pre‐ and post‐treatment differential genes, we advanced to a deeper immune bank sequencing analysis of the tumor tissue. Significant reprogramming of macrophage subpopulations was then discovered after Pc@Zr‐MOF‐mediated SDT. The top six differential genes before and after treatment were labeled to show the effect of SDT on macrophage reprogramming (Figure 5a). Through gene expression profiling, we identified three major macrophage subsets: *Igfbp4*, *Ccl8*, and the *Tgfbi* subgroup (Figure 5b). Subsequently, we fully studied the M1‐type polarization trend of macrophage subpopulation and studied several different outcomes of cell differentiation by means of the quasi‐temporal method (Figures 5c–e and Supplementary Figure S9). The proportion of M1‐type macrophages significantly increased, characterized by high expression of pro‐inflammatory cytokines (e.g., *IL12*, *TNF*) and antigen‐presenting molecules (e.g., HLA‐DR, *CD86)*, indicating enhanced anti‐tumor activity. Concurrently, the proportion of M2‐type macrophages decreased markedly, with downregulation of their signature genes (e.g., *ARG1*, *CD163*), suggesting that Pc@Zr‐MOF suppresses the immunosuppressive state in the tumor microenvironment.

Our pseudo‐temporal trajectory analysis revealed dynamic reprogramming of macrophage phenotypes following Pc@Zr‐MOF‐mediated SDT. The cells underwent bifurcated differentiation: one branch progressing toward pro‐inflammatory M1 polarization, while the other diverged from immunosuppressive M2 states. Notably, transitional‐state macrophages exhibited unique metabolic rewiring—characterized by enhanced glycolytic flux and mitochondrial ROS production—providing novel mechanistic insights into SDT‐induced immunomodulation. These findings collectively demonstrate how acoustic energy conversion directs innate immune cell differentiation through both transcriptional and metabolic axes. Further functional enrichment analysis showed that M1‐type macrophages were significantly enriched in immune activation‐related pathways (e.g., NF‐κB signaling), while M2‐type macrophages were associated with angiogenesis and matrix remodeling pathways. These results demonstrate that Pc@Zr‐MOF promotes a shift from an immunosuppressive to an immunostimulatory tumor microenvironment by regulating macrophage reprogramming and polarization.

Further immunofluorescence analysis of tumor tissues after treatment showed that the expression of antigen‐presenting marker *CD1c* and T cell activation marker *CD80* in HCC tissues was significantly increased after enhanced SDT (Figure 5f). This suggests that polarized M1 cells and antigen‐presenting cells effectively reverse the local tumor microenvironment, making cold tumor antigen presentation more efficient, and thus activating T lymphocytes. The pseudo‐time series analysis of differential genes in the macrophage subpopulation showed that during the reprogramming of macrophages, the expression of genes related to antigen presentation tended to be overexpressed, while the expression of suppressor genes was suppressed, which was consistent with the above results (Figure 5g). Macrophage metabolic reprogramming plays a key role in reversing the tumor microenvironment and activating T cells during enhanced sonodynamic therapy mediated by Pc@Zr‐MOF, resulting in long‐term anti‐tumor immunity in tumor‐bearing mice. However, whether the sonodynamic role of macrophages can be truly transformed into a killing effect on tumor cells remains to be further analyzed, and whether it can induce differentiation of T lymphocytes and promote local infiltration remains unknown. We are hopeful that the combined SDT treatment using MOF and Zn‐Pc will effectively enhance antigen presentation and macrophage activation, thereby improving the immune microenvironment tolerance of the tumor. Therefore, in the future, we will further verify the immune activation effect from this perspective.

**FIGURE 6:**
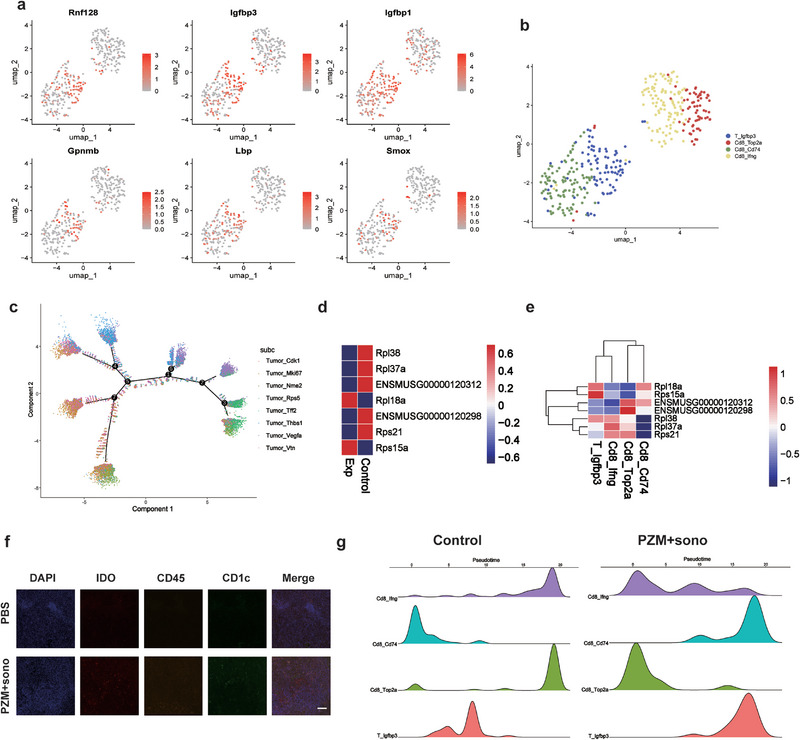
T lymphocyte cluster analysis of Pc@Zr‐MOF sonodynamic therapy in single‐cell immune repertoire. (a, b) Annotated map of the top six differential gene annotations and major T cell subpopulations in umap distribution, (c) The main pseudotime differentiation direction of tumor cells under the influence of immune cells, (d, e) Differential gene heat maps of T cells and their major subpopulations before and after SDT, (f) Immunofluorescence images of tumor sections in the primary treatment group, including IDO, CD45, CD1c, and DAPI staining. Scale: 200 µm, and (g) Comparison of differentiation time of major T cell subsets by quasi‐temporal analysis.

Therefore, in order to further understand the changes and infiltration of T cells in the tumor microenvironment, we annotated and compared the TCR‐related cells. T lymphocytes were annotated by the UMAP distribution into four subgroups as shown in the figure, and the top six differential genes were labeled in the T lymphocyte subgroup (Figures 6a, b). The pseudo‐time series analysis of cell differentiation revealed that the end point of T cell differentiation was much more complex than that of macrophages, and tumor cells also showed a tendency of multiple differentiation under the action of multiple immune cells (Figure 6c). Careful comparison of transcriptome expression differential genes before and after treatment in T cells and subgroups showed that ribosome‐related genes showed a downregulation trend, which may be one of the main ways that enhanced sonodynamic therapy inhibits tumor differentiation and proliferation (Figures 6d, e). In fact, cytotoxic T cells, mainly CD8^+^ T cells, showed a trend of decline between the proliferation of differentiated tumor cells. Further immunofluorescence section analysis of tumor tissues showed that DC cells were regulated and activated by macrophages and T cells and then enriched in tumor tissues to help the body obtain long‐term anti‐tumor immunity (Figure 6f). Through the pseudotime analysis of the differentiation time of T cell subsets, it was found that CD8+ T cells showed an earlier differentiation trend under the stimulation of SDT, while regulatory T cells showed a later differentiation trend, which further verified the previous results. The results were verified by flow cytometry, and the changes of cell clusters were the same as those of sequencing (Supplementary Figures S11, 12). By comparing the single‐cell immune pool in tumor tissues before and after combined therapy, we found and identified three different CD8+ T cell subsets and revealed the differentiation rule of T cells during the sonodynamic effect.

### 2.5 Differential Gene Enrichment Analysis and Cell Communication Interaction Induced by ROS

**FIGURE 7:**
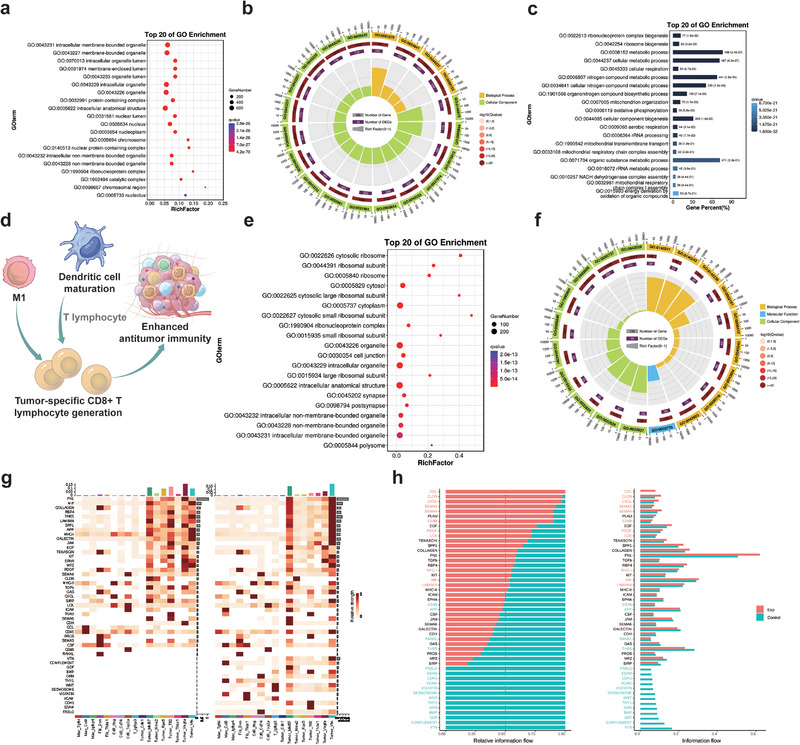
Differential gene enrichment analysis and cell communication analysis were performed before and after Pc@Zr‐MOF sonodynamic therapy. (a, b) The GO enrichment analysis of differential genes in tumor cells mainly listed the top 20 enrichment pathways, (c) GO enrichment trees show changes in the communication pathways inside and outside cells, (d) The schematic diagram reveals the influencing factors of local long‐term immunity in tumor tissue, (e, f) The GO enrichment analysis of differential genes in T cells mainly listed the top 20 enrichment pathways, and (g, h) Cell communication characteristic gene changes in tumor subsets before and after Pc@Zr‐MOF sonodynamic treatment.

By analyzing the enrichment pathways of differential genes in SDT, we observed that ROS primarily influence the expression of organelle‐related genes within HCC tissues, which is consistent with the above results (Figures 7a, b). Specifically, ROS‐mediated regulation of cytoplasmic protein expression plays a critical role in modulating intracellular and extracellular communication, ultimately affecting tumor cell differentiation and proliferation (Figure 7c). This regulatory mechanism highlights the dual role of ROS in not only directly damaging tumor cells but also reshaping the cellular microenvironment to suppress tumor progression. Furthermore, the activation of antigen‐presenting cells (APCs), including M1 macrophages and activated dendritic cells (DCs), was found to play a pivotal role in sustaining anti‐tumor immunity. These APCs enhance the efficacy of ROS‐based SDT by activating cytotoxic T cells, thereby amplifying the immune response against tumor cells (Figure 7d, Supplementary Figure S13). This interplay between ROS and immune cells underscores the potential of SDT to synergize with the immune system for improved therapeutic outcomes.

Enrichment analysis further revealed that ROS predominantly affects T cells by modulating ribosome‐related genes, which are crucial for promoting the differentiation and maturation of CD8+ T cells (Figures 7e, f). This finding suggests that ROS not only directly targets tumor cells but also enhances the adaptive immune response by facilitating the development of cytotoxic T cells. Additionally, differences in cell communication patterns before and after SDT were observed, revealing distinct differentiation trends among tumor cell subsets. Tumor cell subsets with a tendency toward apoptosis exhibited active cell communication, while other subsets displayed a programmed cell death phenotype (Figures 7g, h). These observations indicate that SDT induces heterogeneous responses within the tumor cell population, with some subsets undergoing apoptosis and others entering a state of programmed death. This dynamic shift in cell communication and differentiation further emphasizes the multifaceted impact of ROS in driving tumor regression and enhancing therapeutic efficacy.

## 3 Discussion

HCC tumors have significant internal heterogeneity and suppression of the immune microenvironment, resulting in differences in immunotherapy responses and low reactivity [35, 36]. Although the strategy of SDT has been introduced into the precision treatment of tumors in recent years, there are still some unsolved problems and mechanisms that need to be explained [37, 38, 39]. The development of Pc@Zr‐MOF reshapes the differentiation of cell subsets and the tumor microenvironment in synergistic SDT for HCC. Our findings demonstrate that this novel hybrid material not only enhances the generation of ROS but also modulates the tumor immune microenvironment, leading to improved therapeutic outcomes. The ability of Pc@Zr‐MOF to promote M1 macrophage polarization and cytotoxic T cell activation highlights its potential to overcome the immunosuppressive nature of HCC. This dual mechanism of action, combining direct tumor cell killing with immune modulation, offers a promising strategy for addressing the limitations of current therapies.

To comprehensively elucidate the dynamic effects of ROS generation on cellular redistribution and transcriptional reprogramming within the tumor microenvironment during SDT, we employed scRNA‐seq as a powerful analytical tool [40, 41, 42, 43]. This high‐resolution approach enabled us to capture the nuanced cellular responses and intercellular communication networks modulated by Pc@Zr‐MOF‐mediated SDT in unprecedented detail. The scRNA‐seq data revealed profound remodeling of the tumor immune landscape, characterized by three key alterations: (1) an increase in tumor‐infiltrating cytotoxic CD8+ T cells, (2) enhanced activation markers in NK cells, and (3) a significant reduction in immunosuppressive Tregs and M2‐polarized tumor‐associated macrophages. These coordinated changes indicate a fundamental transition from an immunosuppressive to an immunostimulatory tumor microenvironment, which is essential for establishing immunological memory and achieving durable tumor control. Particularly noteworthy was the observed repolarization of macrophage subsets, with the M1/M2 ratio increasing post‐treatment, demonstrating Pc@Zr‐MOF's unique capacity to reverse tumor‐promoting inflammation and establish an anti‐tumorigenic microenvironment.

Recent advances in MOF‐based SDT have primarily focused on improving ROS yields [24], whereas our Pc@Zr‐MOF system uniquely couples efficient sonosensitization with single‐cell‐resolved immune reprogramming—an underexplored dimension in the field. Unlike conventional porphyrin‐MOFs that show limited hypoxia adaptability, our design integrates catalase‐like activity and metabolic modulation to overcome tumor‐specific barriers. This multimodal approach establishes a new paradigm for SDT platforms, where material properties and immunological outcomes are co‐optimized through mechanistic understanding. Our Pc@Zr‐MOF design rationale stems from the need to overcome two key limitations in HCC treatment: hypoxia‐induced therapy resistance and insufficient immune activation. Compared to previous MOF‐based sonosensitizers like porphyrin‐MOFs, our system demonstrates superior depth penetration due to the optimized acoustic properties of Zr‐MOF and deeper tissue penetration of Zn‐Pc's red‐shifted absorption. This platform shows strong potential for combination with immune checkpoint inhibitors or anti‐angiogenic therapies, as evidenced by the increase in CD8+ T cell infiltration. Importantly, this work advances the SDT field by being the first to integrate single‐cell‐resolution immune profiling with MOF‐based sonosensitizer design, setting a new standard for mechanistic understanding of SDT‐induced immune modulation. The attenuation characteristics of biological tissues may limit treatment effectiveness for deeply seated tumors, particularly in patients with complex anatomical considerations. These challenges may be addressed through emerging technical solutions, including image‐guided transducer positioning, optimized frequency selection based on tumor depth, and contrast‐enhanced sonoporation techniques [44]. Furthermore, the heterogeneous vascularization of advanced HCC lesions could influence nanomaterial distribution patterns, suggesting the potential utility of fractionated dosing regimens or combinatorial vascular normalization approaches.

To translate these promising preclinical findings into clinical applications, future investigations should systematically address several critical aspects [45, 46, 47]. First, pharmacokinetic studies are needed to optimize the nanoparticle delivery parameters, including dosage regimens, administration routes, and ultrasound activation protocols (e.g., intensity, duration, and frequency). Second, longitudinal studies in immunocompetent models should evaluate the treatment's durability, monitoring for potential tumor escape mechanisms and assessing memory immune cell formation. The therapeutic synergy between Pc@Zr‐MOF and existing clinical modalities, particularly immune checkpoint inhibitors (anti‐PD‐1/PD‐L1) or tyrosine kinase inhibitors (sorafenib/lenvatinib), warrants thorough investigation through carefully designed combination studies. The Pc@Zr‐MOF strategy may benefit other hypoxic tumors (e.g., pancreatic/glioblastoma cancers) where immunosuppression limits therapy, though anatomical barriers may require parameter optimization. Importantly, comprehensive toxicology assessments and good laboratory practice studies must precede clinical translation to ensure patient safety. Beyond demonstrating preclinical efficacy, this work establishes a new paradigm for engineering immuno‐active nanomaterials that bridge physical energy conversion and systemic immune awakening. These research directions, combined with our current findings, position Pc@Zr‐MOF as a multifaceted therapeutic platform capable of addressing the dual challenges of direct tumor eradication and immune microenvironment reprogramming in HCC, ultimately blurring the boundaries between materials science and precision immuno‐oncology.

## 4 Conclusion

This study demonstrates the transformative potential of Pc@Zr‐MOF as a novel SDT agent for HCC, combining the unique properties of phthalocyanine and metal‐organic frameworks to enhance ROS generation under ultrasound activation while reprogramming the tumor immune microenvironment. By addressing critical challenges of hypoxia‐induced resistance and immunosuppression, Pc@Zr‐MOF achieves a dual mechanism of action, direct tumor cell killing and immune cell reprogramming, that reshapes the tumor microenvironment into a more immunostimulatory state through M1 macrophage polarization, cytotoxic T cell infiltration, and suppression of immunosuppressive cell populations. This work not only advances the field of SDT but also opens new avenues for combining it with immunomodulatory strategies, such as immune checkpoint inhibitors or adoptive cell therapies, to amplify anti‐tumor immunity. Future research should focus on optimizing delivery and activation parameters, evaluating long‐term efficacy and safety in clinical trials, and leveraging insights from scRNA‐seq to explore applications in other solid tumors. Ultimately, this study represents a significant step toward personalized, multimodal cancer treatments that harness both physical and biological mechanisms to achieve durable therapeutic outcomes, offering new hope for patients with advanced HCC and beyond.

## Author Contributions

**Han Wu**: writing – review and editing, writing – original draft, validation, methodology, investigation, formal analysis, data curation, conceptualization. **Lihui Gu**: writing – review and editing, validation, methodology, formal analysis, data curation, investigation. **Jiahao Xu**: writing – review and editing, validation, methodology, investigation, formal analysis, data curation. **Chuyue Zhang**: writing – review and editing, validation, methodology, formal analysis, data curation. **Mingda Wang**: writing – review and editing, validation, methodology, investigation, formal analysis. **Chao Li**: writing – review and editing, validation, methodology, formal analysis, resources. **Lanqing Yao**: writing – review and editing, validation, methodology, resources, investigation. **Yongkang Diao**: writing – review and editing, validation, methodology, formal analysis, data curation. **Yuchen Li**: writing – review and editing, validation, methodology, investigation, data curation. **Fujie Chen**: writing – review and editing, validation, methodology, formal analysis, data curation. **Huixuan Fan**: writing – review and editing, validation, methodology, investigation, data curation. **Yuze Zhao**: writing – review and editing, validation, methodology, formal analysis. **Feng Shen**: writing – review and editing, validation, supervision, resources, methodology, funding acquisition, conceptualization. **Tian Yang**: writing – review and editing, writing – original draft, validation, supervision, methodology, funding acquisition, formal analysis, data curation, conceptualization.

## Ethics Statement

Animal experiments were carried out in accordance with the Regulations on the Management of Laboratory Animals issued by the Ministry of Science and Technology of the People's Republic of China. This study was approved by the Research Ethics Committee of the Third Affiliated Hospital of Naval Medical University (EDWLL‐034).

## Conflicts of Interest

The authors declare no conflict of interest.

## Supporting information



Supporting Information is available from the journal official website or the corresponding author.
**Supporting File**: exp270151‐sup‐0001‐SuppMat.docx.

## Data Availability

The data that support the findings of this study are available from the corresponding author upon reasonable request.

## References

[1] A. G. Singal , F. Kanwal , and J. M. Llovet , “Global Trends in Hepatocellular Carcinoma Epidemiology: Implications for Screening, Prevention and Therapy,” Nature Reviews Clinical Oncology 20, no. 12 (2023): 864–884, 10.1038/s41571-023-00825-3. PMID: 37884736. 37884736

[2] G. Jia , P. He , T. Dai , et al., “Spatial Immune Scoring System Predicts Hepatocellular Carcinoma Recurrence,” Nature (Early View) (2025): 1–11, 10.1038/s41586-025-08668-x. 40074893

[3] R. Su , X. Tao , L. Yan , et al., “Early Screening, Diagnosis and Recurrence Monitoring of Hepatocellular Carcinoma in Patients With Chronic Hepatitis B Based on Serum N‐glycomics Analysis: A Cohort Study,” Hepatology (Early View) (2025): 10–1097, 10.1097/HEP.0000000000001316. PMC12700682 40117651

[4] D. I. Tsilimigras , R. Kurzrock , and T. M. Pawlik , “Molecular Testing and Targeted Therapies in Hepatobiliary Cancers,” JAMA Surgery ahead of print, February 12, 2025, 10.1001/jamasurg.2025.0242.PMID: 40105823. 40105823

[5] Z. Lin , W. Wang , Y. Yan , Z. Ma , Z. Xiao , and K. Mao , “A Deep Learning‐based Clinical‐radiomics Model Predicting the Treatment Response of Immune Checkpoint Inhibitors (ICIs)‐based Conversion Therapy in Potentially Convertible Hepatocelluar Carcinoma Patients: A Tumor Marker Prognostic Study,” International Journal of Surgery 111, no. 5 (2025): 3342–3355, 10.1097/JS9.0000000000002322. 40085751 PMC12165573

[6] D. L. Yang , L. Ye , F. J. Zeng , et al., “Global Burden of Primary Liver Cancer in 2020 and Predictions to 2040,” Hepatology ahead of print. January 8, 2025, 10.1097/HEP.0000000000001229. PMC9670241 36208844

[7] L. A. Dawson , K. A. Winter , J. J. Knox , et al., “Stereotactic Body Radiotherapy vs. Sorafenib Alone in Hepatocellular Carcinoma,” JAMA Oncology 11, no. 2 (2025): 136–144, 10.1001/jamaoncol.2024.5403. 39699905 PMC11843352

[8] M. Xi , Z. Yang , L. Hu , et al., “Radiofrequency Ablation versus Stereotactic Body Radiotherapy for Recurrent Small Hepatocellular Carcinoma: A Randomized, Open‐Label, Controlled Trial,” Journal of Clinical Oncology 43, no. 9 (2025): 1073–1082, 10.1200/JCO-24-01532. 39693584

[9] C. Qi , L. Shen , T. Andre , et al., “Efficacy and Safety of larotrectinib in Patients With TRK Fusion Gastrointestinal Cancer,” European Journal of Cancer 220 (2025): 115338, 10.1016/j.ejca.2025.115338. 40068370 PMC12517377

[10] N. Wang , S. Lu , Z. Cao , et al., “Pyruvate Metabolism Enzyme DLAT Promotes Tumorigenesis by Suppressing Leucine Catabolism,” Cell Metabolism 37, no. 6 (2025): 1381–1399, 10.1016/j.cmet.2025.02.008. 40112809

[11] M. Lim , M. Espinoza , Y. H. Huang , J. Franses , H. Zhu , and D. Hsiehchen , “Complete Response to Immunotherapy in Patients with Hepatocellular Carcinoma,” JAMA Network Open 8, no. 2 (2025): e2461735, 10.1001/jamanetworkopen.2024.61735. PMID: 39998829. 39998829 PMC11862977

[12] Y. Myojin , S. Babaei , R. Trehan , et al., “Evolutionary Trajectories of Hepatocellular Carcinoma Under Multi‐Layered Selection Pressures,” Gut ahead of print, 2025, 10.1136/gutjnl-2024-334026.

[13] J. Cai , P. Zhang , Y. Cai , et al., “Lactylation‐Driven NUPR1 Promotes Immunosuppression of Tumor‐Infiltrating Macrophages in Hepatocellular Carcinoma,” Advanced Science 12, no. 20 (2025): e2413095, 10.1002/advs.202413095. 40305758 PMC12120759

[14] Y. Lu , L. Xu , W. Chen , et al., “Intrahepatic Microbial Heterogeneity in Multifocal Hepatocellular Carcinoma and Its Association With Host Genomic and Transcriptomic Alterations,” Cancer Discovery ahead of print, January 14, 2025, 10.1158/2159-8290.CD-24-1259. PMC12319405 40287964

[15] D. K. Chiu , X. Zhang , B. Y. Cheng , et al., “Tumor‐derived Erythropoietin Acts as an Immunosuppressive Switch in Cancer Immunity,” Science 388, no. 6745 (2025): eadr3026, 10.1126/science.adr3026. 40273234 PMC12110762

[16] J. Zheng , F. Zhao , E. Pariente , et al., “Tumor‐Targeted Glutamine Metabolism Blocker Synergizes With *TiO_2_ *‐Au Janus Nanoparticles for Enhanced Sono‐Metabolic Antitumor Therapy,” Advanced Materials (Early View) (2025): e2418800, 10.1002/adma.202418800. 39950402

[17] D. Y. Hou , Q. You , P. Zhang , et al., “Cascade‐Activatable Nanoprodrug System Augments Sonochemotherapy of Bladder Cancer,” ACS Nano 18, no. 52 (2024): 35507–35519, 10.1021/acsnano.4c12967. 39686741

[18] D. Wen , J. Feng , R. Deng , K. Li , and H. Zhang , “Zn/Pt Dual‐site Single‐atom Driven Difunctional Superimposition‐augmented Sonosensitizer for Sonodynamic Therapy Boosted Ferroptosis of Cancer,” Nature Communications 15, no. 1 (2024): 9359, 10.1038/s41467-024-53488-8. PMID: 39472589. PMC11522694 39472589

[19] Y. Xu , Y. Pang , L. Luo , et al., “De Novo Designed Ru(II) Metallacycle as a Microenvironment‐Adaptive Sonosensitizer and Sonocatalyst for Multidrug‐Resistant Biofilms Eradication,” Angewandte Chemie (International Edition in English) 63, no. 15 (2024): e202319966, 10.1002/anie.202319966. 38327168

[20] X. Qu , F. Yin , M. Pei , et al., “Modulation of Intratumoral *Fusobacterium nucleatum* to Enhance Sonodynamic Therapy for Colorectal Cancer With Reduced Phototoxic Skin Injury,” ACS Nano 17, no. 12 (2023): 11466–11480, 10.1021/acsnano.3c01308. 37201179 PMC10311605

[21] Q. Wang , Y. Wen , B. Bi , et al., “Oxygen/Sulfate Radicals‐generating *CaS_2_O_8_ * Nanosonosensitizers Induce PANoptosis and Calcium Overload for Enhanced Peritoneal Metastasis Immunotherapy,” Science Bulletin ahead of print, March 5, 2025, 10.1016/j.scib.2025.03.015. 40118724

[22] L. Yan , L. Chang , Y. Tian , et al., “Graphene Quantum Dot Sensitized Heterojunctions Induce Tumor‐Specific Cuproptosis to Boost Sonodynamic and Chemodynamic Enhanced Cancer Immunotherapy,” Advanced Science 12, no. 7 (2025): e2410606, 10.1002/advs.202410606. 39716968 PMC11831527

[23] F. Wang , Y. Fan , Y. Liu , et al., “Oxygen‐Carrying Semiconducting Polymer Nanoprodrugs Induce Sono‐Pyroptosis for Deep‐Tissue Tumor Treatment,” Exploration 4, no. 4 (2024): 20230100, 10.1002/EXP.20230100. 39175882 PMC11335461

[24] X. Pan , Z. Huang , J. Guo , et al., “MOF‐Derived Nanoparticles With Enhanced Acoustical Performance for Efficient Mechano‐Sonodynamic Therapy,” Advanced Materials 36, no. 33 (2024): e2400142, 10.1002/adma.202400142. 38896775

[25] L. Cai , J. Du , F. Han , et al., “Piezoelectric Metal–Organic Frameworks Based Sonosensitizer for Enhanced Nanozyme Catalytic and Sonodynamic Therapies,” ACS Nano 17, no. 8 (2023): 7901–7910, 10.1021/acsnano.3c01856. 37052950

[26] X. Meng , S. Sun , C. Gong , et al., “Ag‐Doped Metal–Organic Frameworks′ Heterostructure for Sonodynamic Therapy of Deep‐Seated Cancer and Bacterial Infection,” ACS Nano 17, no. 2 (2022): 1174–1186, 10.1021/acsnano.2c08687. 36583572

[27] X. Pan , W. Wang , Z. Huang , et al., “MOF‐Derived Double‐Layer Hollow Nanoparticles With Oxygen Generation Ability for Multimodal Imaging‐Guided Sonodynamic Therapy,” Angewandte Chemie (International Edition in English) 59, no. 32 (2020): 13557–13561, 10.1002/anie.202004894. 32374941

[28] C. Xu , J. Dong , X. Shi , et al., “Engineered Microalgae for Photo‐sonodynamic Synergistic Therapy in Breast Cancer Treatment,” Acta Biomaterialia 193 (2025): 531–544, 10.1016/j.actbio.2024.12.047. 39709158

[29] C. Zhang , L. Xin , J. Li , et al., “Metal–Organic Framework (MOF)‐Based Ultrasound‐Responsive Dual‐Sonosensitizer Nanoplatform for Hypoxic Cancer Therapy,” Advanced Healthcare Materials 11, no. 2 (2022): e2101946, 10.1002/adhm.202101946. 34706160

[30] Y. Bao , J. Chen , H. Qiu , et al., “Erythrocyte Membrane‐Camouflaged PCN‐224 Nanocarriers Integrated With Platinum Nanoparticles and Glucose Oxidase for Enhanced Tumor Sonodynamic Therapy and Synergistic Starvation Therapy,” ACS Applied Materials and Interfaces 13, no. 21 (2021): 24532–24542, 10.1021/acsami.1c05644. 34019368

[31] A. Turkkol , C. Can Karanlik , S. G. Caliskan , M. D. Bilgin , A. Erdogmus , and E. Guzel , “Hybrid Sono‐Photodynamic Combination Therapy Mediated by Water‐Soluble Gallium Phthalocyanine Enhances the Cytotoxic Effect Against Breast Cancer Cell Lines,” ACS Applied Bio Materials 7, no. 5 (2024): 2725–2733, 10.1021/acsabm.3c01078. 38591733

[32] L. C. Nene and H. Abrahamse , “Design Consideration of Phthalocyanines as Sensitizers for Enhanced Sono‐photodynamic Combinatorial Therapy of Cancer,” Acta Pharmaceutica Sinica B 14, no. 3 (2024): 1077–1097, 10.1016/j.apsb.2023.11.030. 38486981 PMC10935510

[33] P. H. Zhao , Y. L. Wu , X. Y. Li , et al., “Aggregation‐Enhanced Sonodynamic Activity of Phthalocyanine–Artesunate Conjugates,” Angewandte Chemie (International Edition in English) 61, no. 5 (2022): e202113506, 10.1002/anie.202113506. 34761489

[34] J. H. Cavka , S. Jakobsen , U. Olsbye , et al., “A New Zirconium Inorganic Building Brick Forming Metal Organic Frameworks With Exceptional Stability,” Journal of the American Chemical Society 130, no. 42 (2008): 13850–13851, 10.1021/ja8057953. 18817383

[35] C. C. Wong and C. M. Wong , “ETV5‐S100A9 feed‐forward Loop Connecting HCC and MDSCs to Shape the Immunosuppressive Tumour Microenvironment,” Gut ahead of print, March 12, 2025, 10.1136/gutjnl-2025-335078. 40147932

[36] J. Pan , M. Zhang , D. Rao , et al., “CAD Manipulates Tumor Intrinsic DHO/UBE4B/NF‐κB Pathway and Fuels Macrophage Cross‐talk, Promoting HCC Metastasis,” Hepatology ahead of print, March 12, 2025, 10.1097/HEP.0000000000001304. 40073276

[37] K. Wang , L. Li , G. Liang , H. Xiao , L. Zhang , and T. Liu , “Sonodynamic Activated Nanoparticles With Glut1 Inhibitor and Cystine‐containing Polymer Stimulate Disulfidptosis for Improved Immunotherapy in Bladder Cancer,” Biomaterials 319 (2025): 123178, 10.1016/j.biomaterials.2025.123178. 39978048

[38] Z. Chen , L. Sang , Y. Liu , and Z. Bai , “Sono‐Piezo Dynamic Therapy: Utilizing Piezoelectric Materials as Sonosensitizer for Sonodynamic Therapy,” Advanced Science (2025): e2417439, 10.1002/advs.202417439. 39921482 PMC11948011

[39] Z. Quan , S. Wang , H. Xie , et al., “ROS Regulation in CNS Disorder Therapy: Unveiling the Dual Roles of Nanomedicine,” Small 21, no. 5 (2025): e2410031, 10.1002/smll.202410031. 39676433

[40] H. Peng , D. Wang , S. Huang , and A. Yu , “Dual‐targeting Aggregation‐induced Emission Polymer Micelles Mediate Immunogenic Sonodynamic Therapy for Tumor Cell Growth Inhibition and Macrophage Reprogramming,” Acta Biomaterialia 195 (2025): 321–337, 10.1016/j.actbio.2025.01.065. 39900272

[41] G. D. Lewis , G. Li , J. Guo , et al., “The HER2‐directed Antibody‐drug Conjugate DHES0815A in Advanced and/or Metastatic Breast Cancer: Preclinical Characterization and Phase 1 Trial Results,” Nature Communications 15, no. 1 (2024): 466, 10.1038/s41467-023-44533-z. PMC10784567 38212321

[42] J. Liang , X. Qiao , L. Qiu , et al., “Engineering Versatile Nanomedicines for Ultrasonic Tumor Immunotherapy,” Advanced Science 11, no. 3 (2024): e2305392, 10.1002/advs.202305392. 38041509 PMC10797440

[43] M. Li , Y. Liu , Y. Zhang , N. Yu , and J. Li , “Sono‐Activatable Semiconducting Polymer Nanoreshapers Multiply Remodel Tumor Microenvironment for Potent Immunotherapy of Orthotopic Pancreatic Cancer,” Advanced Science 10, no. 35 (2023): e2305150, 10.1002/advs.202305150. 37870196 PMC10724419

[44] D. Wang , L. Ji , Y. Li , et al., “Intrinsic Chiral‐dependent Autophagy for Tumor Ablation,” Nature Nanotechnology ahead of print, January 23, 2025, 10.1038/s41565-025-01943-y.

[45] J. Guo , H. Han , H. Zhao , D. Jia , L. Yin , and J. Sha , “Cascade‐enhanced Based‐polyoxometalates Nanozyme for Glutathione Detection and Tumor Cell Disruption,” Talanta 291 (2025): 127890, 10.1016/j.talanta.2025.127890. 40056648

[46] K. Javed , N. Abbas , M. Bilal , et al., “Fabrication of a *ZnFe_2_O_4_ *@Co/Ni‐MOF Nanocomposite and Photocatalytic Degradation Study of Azo Dyes,” RSC Advances 14, no. 42 (2024): 30957–30970, 10.1039/d4ra05283h. 39346520 PMC11429226

[47] P. Yadav , S. Kumari , A. Yadav , et al., “Biocompatible Drug Delivery System Based on a MOF Platform for a Sustained and Controlled Release of the Poorly Soluble Drug Norfloxacin,” ACS Omega 8, no. 31 (2023): 28367–28375, 10.1021/acsomega.3c02418. 37576664 PMC10413448

