# Supplementary material for: Immunosuppressive Microenvironment Reprogramming by Synergistic Sonodynamic Therapy of Phthalocyanine‐MOF Hybrids for Hepatocellular Carcinoma
Source: Exploration (Beijing). 2026 Feb 22;6(2):20250074. doi: 10.1002/EXP.20250074 (PMC13094521; doi:10.1002/EXP.20250074)
Supplement: Supplementary file 1 — Supporting Information is available from the journal official website or the corresponding author. Supporting File: exp270151‐sup‐0001‐SuppMat.docx. [file EXP2-6-20250074-s001.docx]

Supplementary Materials for

**Synergistic Sonodynamic Therapy and Immune Cell Reprogramming by Phthalocyanine-MOF Hybrids for Hepatocellular Carcinoma**

**This PDF file includes:**

Materials and Methods

Figures. S1 to S13

Materials and Methods

*Synthesis of Pc@Zr-MOF*

Synthesis of Zr-MOF: H_2_TCPP, 100 mg, 0.13 mmol, ZrOCl_2_·8H_2_O, 300 mg, 0.93 mmol, and benzoic acid, 2.9 g, 24 mmol, were dissolved in 100 mL of dimethylformamide, DMF. The mixture was stirred at 90 °C with a rotation speed of 300 rpm for 5 hours. Upon completion of the reaction, the mixture was centrifuged at 10,000 rpm for 10 minutes to collect the nanoparticles, NPs. The obtained NPs were subsequently washed three times with fresh DMF. After modification by DSPE-PEG, the synthesized Zr-MOF was dissolved in water and PBS.

Loading of the Zn-Pc: To prepare the final aqueous solution of Pc@Zr-MOF, 10 mg of Zr-MOF and 10 mg of Zn-Pc were mixed in 5 mL of double-distilled water (ddH2O) and stirred continuously for 24 hours. The resulting Pc@Zr-MOF solution was then stored at 4 °C for further use. The efficiency of drug loading was assessed using ultraviolet-visible-near-infrared (UV-vis-NIR) spectroscopy.

*Characterization of Pc@Zr-MOF*

The structural characteristics of Zr-MOF and Pc@Zr-MOF were investigated using transmission electron microscopy (TEM). Additionally, the crystallographic properties of Pc@Zr-MOF were analyzed through X-ray diffractometry (XRD). The elemental distribution within Pc@Zr-MOF was verified using spectral analysis and element mapping techniques. The dynamic light scattering (DLS) properties and zeta potential of Zr-MOF, Zn-Pc, and Pc@Zr-MOF were measured at 25 °C and pH 7.4 using a particle analyzer (Malvern, England). Furthermore, the functional groups and molecular structure of Pc@Zr-MOF were characterized using infrared spectroscopy and hydrogen nuclear magnetic resonance (NMR) spectroscopy, respectively. Furthermore, we digested the MOF loaded with Zn-Pc using aqua regia, terminated the digestion and filtered it, and then conducted ICP-OES testing. Based on the obtained concentration of Zn element, the content of Zn-Pc on the load is calculated, and then the loading rate of the MOF is determined.

*Stability test and hemolysis essay*

For the stability test, Pc@Zr-MOF was dispersed in aqueous solution and stored at 4 °C and room temperature. Samples were collected at predetermined time intervals (0, 1, 3, 5, and 7 days) to analyze changes in particle size and structural integrity using dynamic light scattering (DLS) and transmission electron microscopy (TEM). For the hemolysis assay, fresh red blood cells (RBCs) were isolated from whole blood and washed with phosphate-buffered saline (PBS). Pc@Zr-MOF was then incubated with RBCs at concentrations ranging from 10 to 100 µg/mL for 2 hours at 37 °C. After centrifugation, the supernatant was collected, and the absorbance at 540 nm was measured to quantify hemoglobin release. PBS and deionized water were used as negative and positive controls, respectively.

*Cytophagocytosis experiment*

For the cytophagocytosis experiment, cells were seeded in 24-well plates and cultured overnight to allow adherence. The cells were then divided into four groups: control, Zr-MOF, Zn-Pc, and Pc@Zr-MOF. Each group was treated with corresponding materials labeled with the fluorescent dye Cy5.5 and incubated for 4 hours at 37 °C in a 5% CO₂ atmosphere. After incubation, the cells were washed three times with phosphate-buffered saline (PBS) to remove uninternalized particles. The cells were fixed with 4% paraformaldehyde for 15 minutes and stained with DAPI to visualize nuclei. Fluorescence microscopy was used to capture images of the cells, with Cy5.5 fluorescence indicating the uptake of the materials. The experiment was performed in triplicate to ensure reproducibility.

*Cell live and death fluorescence staining*

For the live/dead cell assay, cells were seeded in 24-well plates and allowed to adhere overnight. The cells were divided into four groups: control, Zr-MOF, Zn-Pc, and Pc@Zr-MOF. Each group was treated with the corresponding materials and incubated for 24 hours at 37 °C in a 5% CO₂ atmosphere. After incubation, the cells were washed twice with phosphate-buffered saline (PBS) and stained with a live/dead viability kit (e.g., Calcein-AM for live cells and propidium iodide for dead cells) according to the manufacturer’s instructions. The cells were then incubated in the dark at room temperature for 30 minutes. Fluorescence microscopy was used to capture images, with green fluorescence indicating live cells and red fluorescence indicating dead cells. The experiment was performed in triplicate to ensure reproducibility.

*ROS fluorescence quantification assay*

For the ROS fluorescence quantification assay, cells were seeded in 24-well plates and allowed to adhere overnight. The cells were divided into four groups: control, Zr-MOF, Zn-Pc, and Pc@Zr-MOF. Each group was treated with the corresponding materials and incubated for a predetermined time (e.g., 4 or 24 hours) at 37 °C in a 5% CO₂ atmosphere. After incubation, the cells were washed twice with phosphate-buffered saline (PBS) and incubated with the ROS-sensitive fluorescent probe DCFH-DA (10 µM) in serum-free medium for 30 minutes at 37 °C in the dark. The cells were then washed again with PBS to remove excess dye. Fluorescence microscopy was used to capture images, and the fluorescence intensity of DCF (the oxidized product of DCFH-DA) was quantified using image analysis software. The experiment was performed in triplicate to ensure reproducibility.

*In vivo SDT treatment essay*

All animal experiments were conducted with the approval of the Ethics Committee of the Eastern Hepatobiliary Surgery Hospital (Third Affiliated Hospital of Naval Medical University). For the in vivo SDT study, C57 mice bearing Hepa1-6 tumors were randomly divided into four groups: control, Pc@Zr-MOF, PD-L1, and PZM+PD-L1 (n = 5 per group). Tumor volumes were calculated using the formula V = (length × width²) / 2 and monitored every 2 days for 14 days. Mice were administered the corresponding materials via tail vein injection, followed by ultrasound irradiation (1 MHz, 1.5 W/cm², 50% duty cycle) for 10 minutes at the tumor site. Body weight and tumor size were recorded throughout the study. On day 14, mice were euthanized, and tumors were excised to measure final tumor mass. Tumor tissues were collected for further analysis, including histopathological examination, immunohistochemistry, or molecular studies. The experiment was conducted in accordance with ethical guidelines for animal research.

*Immunohistochemistry*

For immunohistochemical analysis, tumor tissues collected from the in vivo study were fixed in 4% paraformaldehyde, embedded in paraffin, and sectioned into 4-µm slices. For TUNEL staining, the sections were deparaffinized, rehydrated, and treated with proteinase K to expose DNA fragments. The TUNEL reaction mixture was applied to label apoptotic cells according to the manufacturer’s instructions, and nuclei were counterstained with DAPI. For Ki-67 staining, the sections were subjected to antigen retrieval, blocked with serum, and incubated with an anti-Ki-67 primary antibody overnight at 4 °C. After washing, the sections were incubated with a secondary antibody conjugated with a fluorescent dye or enzyme (e.g., HRP). The stained sections were visualized under a fluorescence or light microscope, and the percentage of TUNEL-positive (apoptotic) or Ki-67-positive (proliferating) cells was quantified using image analysis software. Each experiment was performed in triplicate to ensure reproducibility.

*Immunofluorescence staining*

For immunofluorescence staining, tumor tissue sections were deparaffinized, rehydrated, and subjected to antigen retrieval. The sections were blocked with 5% bovine serum albumin (BSA) for 1 hour at room temperature to prevent nonspecific binding. Subsequently, the sections were incubated overnight at 4 °C with a mixture of primary antibodies against CD3 (T-cell marker) and CD11b (macrophage marker). After washing with phosphate-buffered saline (PBS), the sections were incubated with species-specific secondary antibodies conjugated to fluorescent dyes (e.g., Alexa Fluor 488 for CD3 and Alexa Fluor 594 for CD11b) for 1 hour at room temperature in the dark. Nuclei were counterstained with DAPI for 10 minutes. Finally, the sections were washed, mounted with antifade medium, and visualized under a fluorescence microscope. Images were captured, and the colocalization of CD3 and CD11b signals was analyzed using image analysis software. Each experiment was performed in triplicate to ensure reproducibility.

*Single-cell RNA sequencing and data processing*

For single-cell RNA sequencing (scRNA-seq) with TCR analysis, tumor tissues from the PD-L1 (Postive Control) and PZM+PD-L1 groups (Experiment) were dissociated into single-cell suspensions using enzymatic digestion and mechanical disruption. The cells were then washed, resuspended in PBS, and subjected to viability staining to ensure >90% viability. Single-cell libraries were prepared using a 10x Genomics Chromium platform according to the manufacturer’s protocol, with specific enrichment for TCR transcripts. Sequencing was performed on an Illumina NovaSeq 6000 platform to obtain high-quality single-cell transcriptomes.

Raw sequencing data were processed using the Cell Ranger pipeline (10x Genomics) to generate gene expression matrices. TCR sequences were extracted and annotated using the Cell Ranger V(D)J pipeline. Low-quality cells (e.g., those with <200 genes or >20% mitochondrial reads) and doublets were removed using the Seurat R package. Gene expression matrices from the Control and Pc@Zr-MOF groups were normalized using the SCTransform method and integrated to correct for batch effects using Seurat’s integration workflow. Principal component analysis (PCA) was performed on highly variable genes, followed by uniform manifold approximation and projection (UMAP) for dimensionality reduction. Cells were clustered using the Louvain algorithm, and clusters were annotated based on canonical marker genes (e.g., CD3E for T cells, CD19 for B cells, CD14 for monocytes).

TCR clonotypes were identified and matched to single-cell transcriptomes. Clonotype expansion and diversity were compared between the Control and Pc@Zr-MOF groups. Differentially expressed genes (DEGs) between the Control and Pc@Zr-MOF groups were identified using the FindMarkers function in Seurat (Wilcoxon rank-sum test, adjusted p-value < 0.05). Gene ontology (GO) and Kyoto Encyclopedia of Genes and Genomes (KEGG) pathway analyses were performed using the clusterProfiler R package to explore functional enrichment. Results were visualized using ggplot2 and pheatmap in R, including UMAP plots, violin plots, and heatmaps of DEGs.

*Macrophage subpopulation analysis*

For macrophage subpopulation analysis, macrophages were subset from the single-cell dataset based on canonical markers (e.g., CD68, CD14) and subclustered using Seurat in R. The top 6 marker genes for each subcluster were identified and annotated, followed by macrophage subpopulation classification into M1-like (e.g., IL1B, NOS2) and M2-like (e.g., CD163, MRC1) phenotypes. M1 and M2 polarization scores were calculated using gene signatures, and pseudotime analysis was performed with Monocle3 to infer dynamic transitions between states. Immunofluorescence staining of tumor tissues with M1 (e.g., iNOS) and M2 (e.g., CD206) markers, along with DAPI nuclear staining, was used to validate scRNA-seq findings. All analyses were conducted in R, with results visualized using ggplot2, pheatmap, and Monocle3.

*T cell subpopulation analysis*

For T cell subpopulation analysis, T cells were subset from the single-cell dataset based on canonical markers (e.g., CD3D, CD3E) and subclustered using Seurat in R. The top 6 marker genes for each subcluster were identified and annotated, followed by T cell subpopulation classification into subsets such as CD8+ T cells (e.g., CD8A, GZMB), CD4+ T cells (e.g., CD4, FOXP3), and regulatory T cells (e.g., IL2RA, CTLA4). CD8+ T cell activity was assessed using a curated gene signature and scored using the `AddModuleScore` function in Seurat. Pseudotime analysis was performed with Monocle3 to infer developmental trajectories and dynamic transitions among T cell states. Immunofluorescence staining of tumor tissues with CD8 (for cytotoxic T cells) and CD4 (for helper T cells) markers, along with DAPI nuclear staining, was conducted to validate scRNA-seq findings. All analyses were performed in R, with results visualized using ggplot2, pheatmap, and Monocle3.

*Cell communication analysis*

For cell-cell interaction and communication analysis, the CellChat R package was employed to infer signaling networks between tumor cells, T cell subpopulations, and macrophage subpopulations. Ligand-receptor pairs were identified, and communication probabilities were calculated to quantify interaction strength. Key enriched pathways included TGF-β signaling (immune suppression), PD-1/PD-L1 signaling (T cell exhaustion), MHC-I/II signaling (antigen presentation), CXCL12-CXCR4 signaling (immune cell recruitment), IL-10 signaling (macrophage polarization), and CD40-CD40L signaling (immune activation). Results were visualized using network plots, heatmaps, and bubble plots, revealing critical communication networks within the tumor microenvironment and their roles in modulating immune responses.

*Statistical analysis*

For statistical analysis, data were processed and analyzed using Origin, GraphPad Prism, and R. Origin was used for data visualization and curve fitting, while GraphPad Prism was employed for statistical comparisons (e.g., t-tests, ANOVA) and generating bar graphs or scatter plots. R was utilized for advanced statistical analyses, including differential gene expression, pathway enrichment, and single-cell data processing. All results were presented as mean ± standard deviation (SD), and statistical significance was defined as p < 0.05. Visualization tools such as ggplot2 in R and Prism’s graphing functions were used to create publication-quality figures.

Supplementary Figures


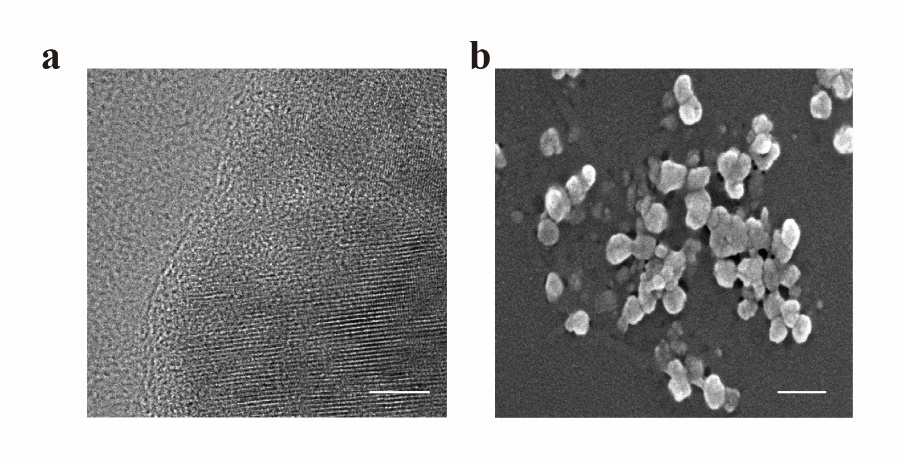


**Figure S1.** TEM and SEM feature images of Pc@Zr-MOF. a) Mainly reflects the lattice fringes of MOF (scale bar: 5 nm). b) SEM image of solution stability test (scale bar: 200 nm).


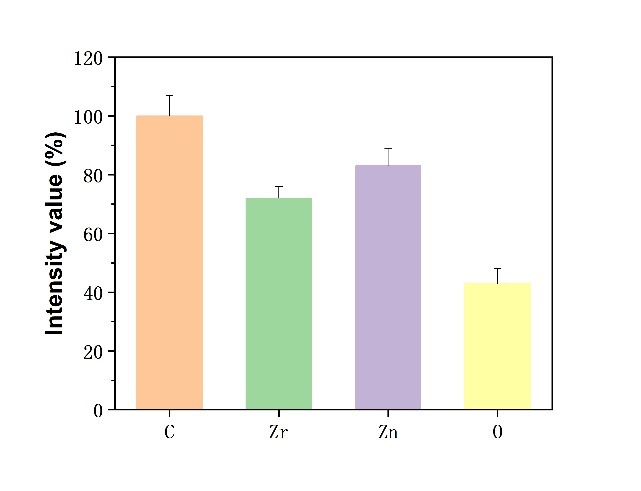


**Figure S2.** Relative quantitative analysis of the scanning results for each element. The quantitative strength of all elements is based on the reference value of carbon, and may be affected by the distribution density and display intensity of the individual elements.


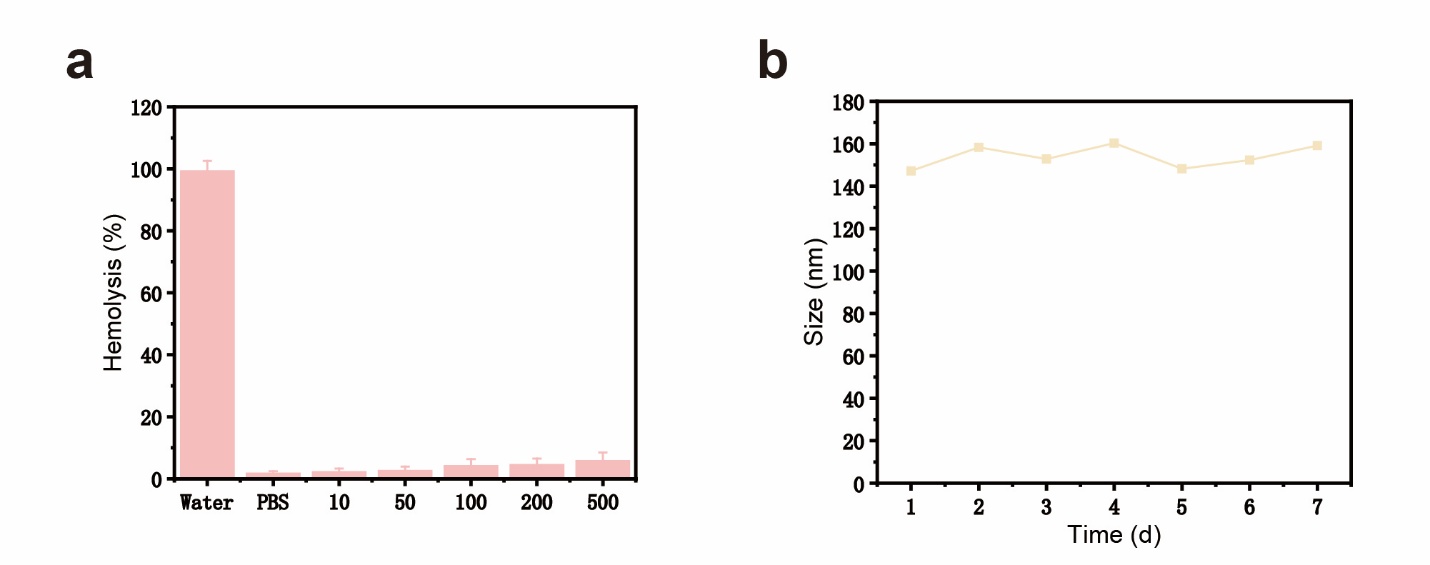


Figure. S3. Hemolysis test and stability test of Pc@Zr-MOF and its derivatives. a) Hemolysis test of Pc@Zr-MOF. b) Stability test of Pc@Zr-MOF.


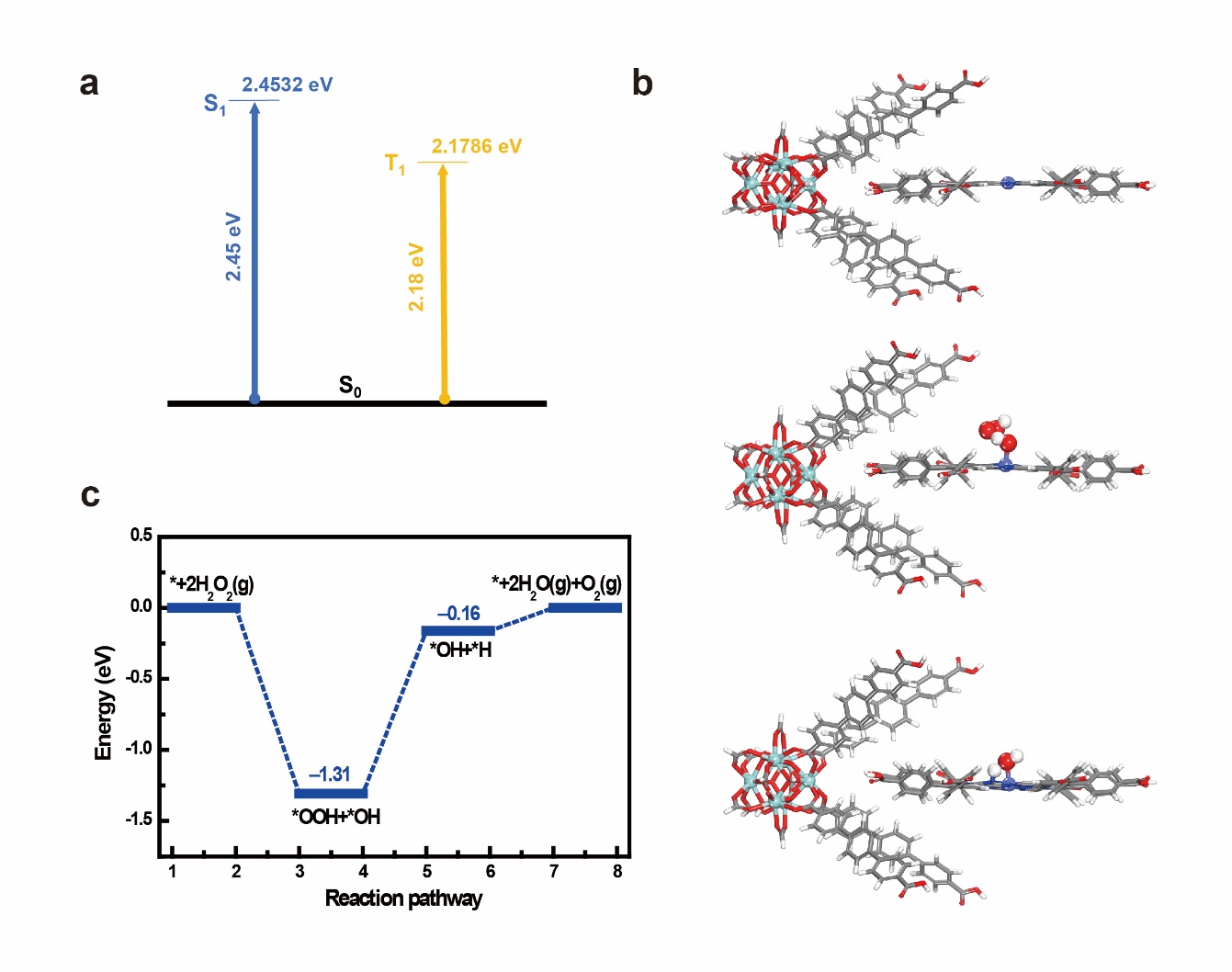


Figure. S4. DFT calculation of catalytic efficiency for Pc@Zr-MOF sonodynamic therapy. a) Calculated energy diagram for T1 of Pc@Zr-MOF in the liquid phases. b) ROS catalytic generation model diagram, the ball-and-stick model shows the molecular site changes in a pore. c) Energy profiles and corresponding reaction pathways for the reaction activities of Pc@Zr-MOF.


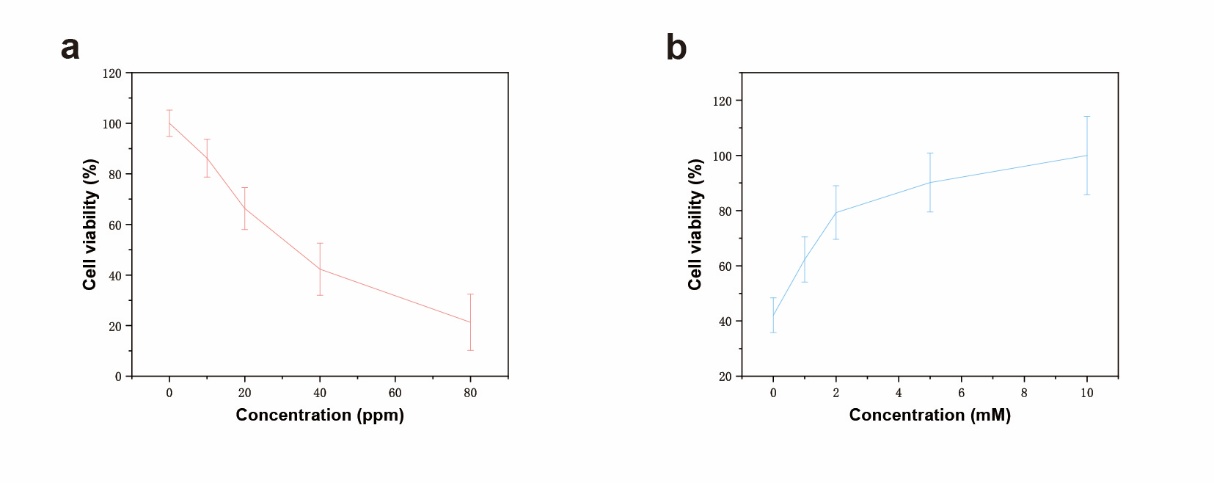


**Figure. S5.** Pc@Zr-MOF mediated relationship between HCC cytotoxicity tests and rescue experiments. a) The relationship between cell activity assay and Pc@Zr-MOF under ultrasound mediation by CCK-8. b) NAC rescue experiment showed that the cells killed ROS source.


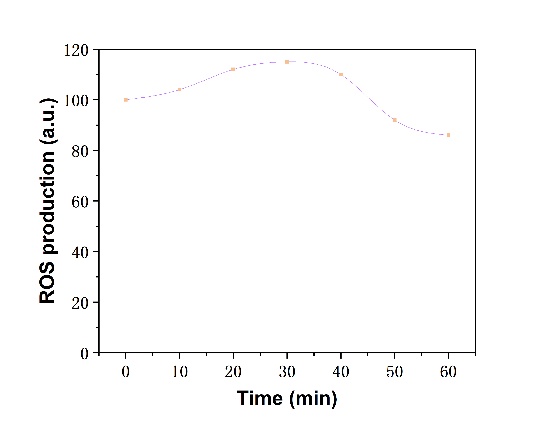


**Figure. S6.** The fitting curves of ROS production changeing over time under the continuous effect of ultrasound. By using the readings from the enzyme analyzer at different time points and using TMB as the ROS capture agent, the stability of ROS production was calculated.


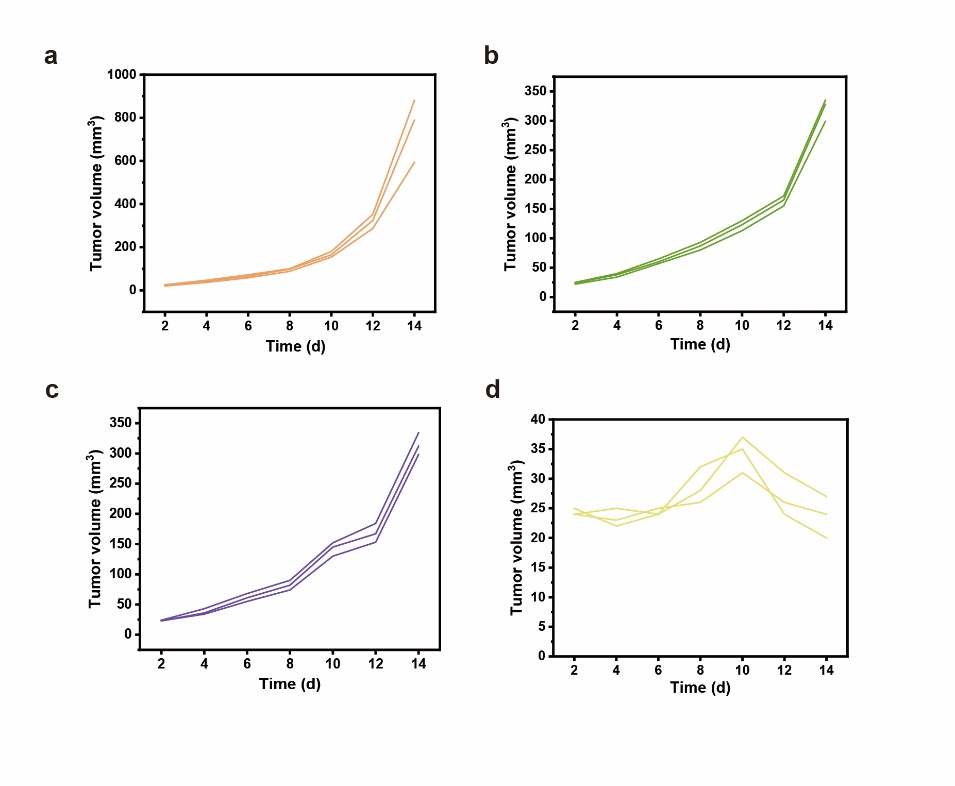


Figure. S7. The growth curves of individual tumors in each group of tumor-bearing mice. Each group of mice was measured for length, width and height on the following day, and the estimated volume was recorded accordingly.


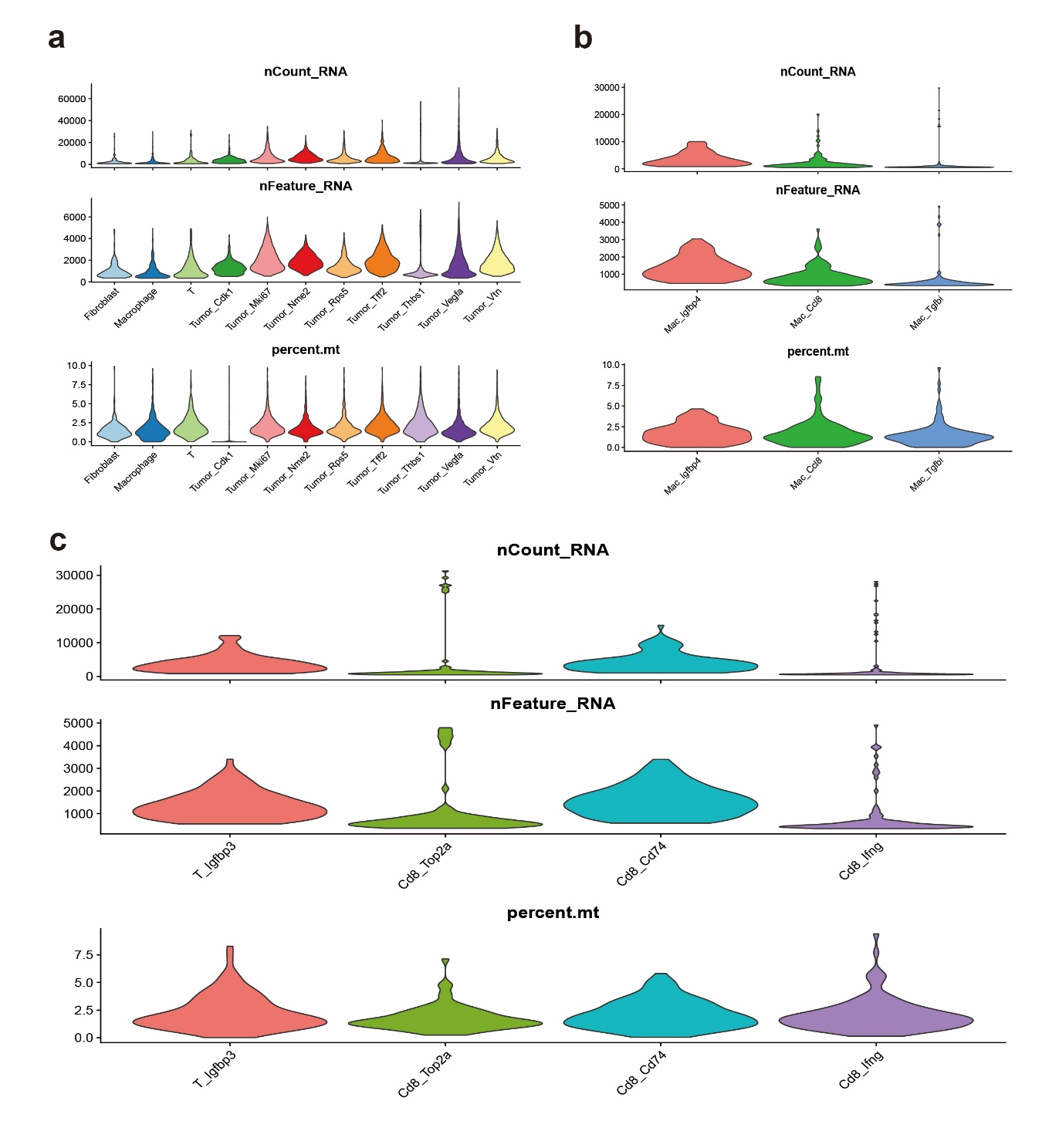


**Figure S8.** RNA quality control of Single cell sequencing for overall and major subgroup. a) Overall cell sequencing quality control. b, c) Sequencing quality data results of macrophages and T cells.


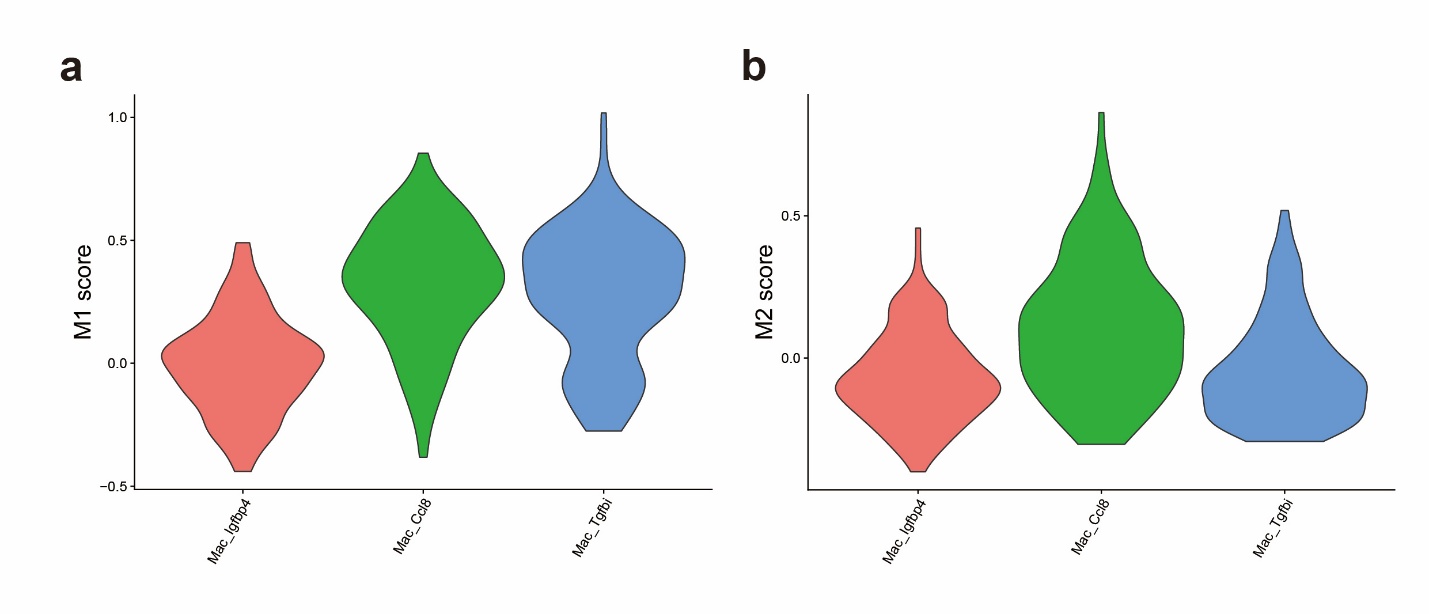


**Figure S9.** Violin distribution map of M1 and M2 scores of each subgroup of macrophages. a) M1 scores of macrophage subgroups. b) M2 scores of macrophage subgroups.


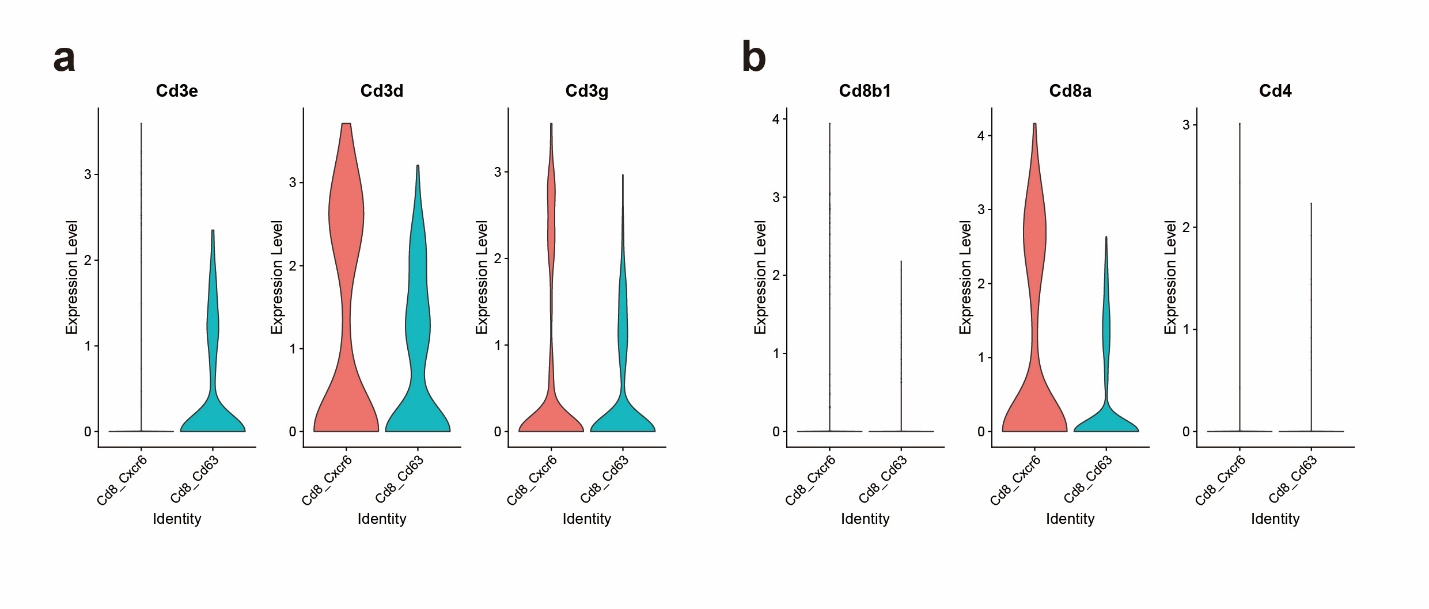


**Figure S10.** The distribution of signature molecules in T cell subsets. a) Distribution of CD3 in T cell subsets. b) Distribution of CD4 and CD8 in T cell subsets.


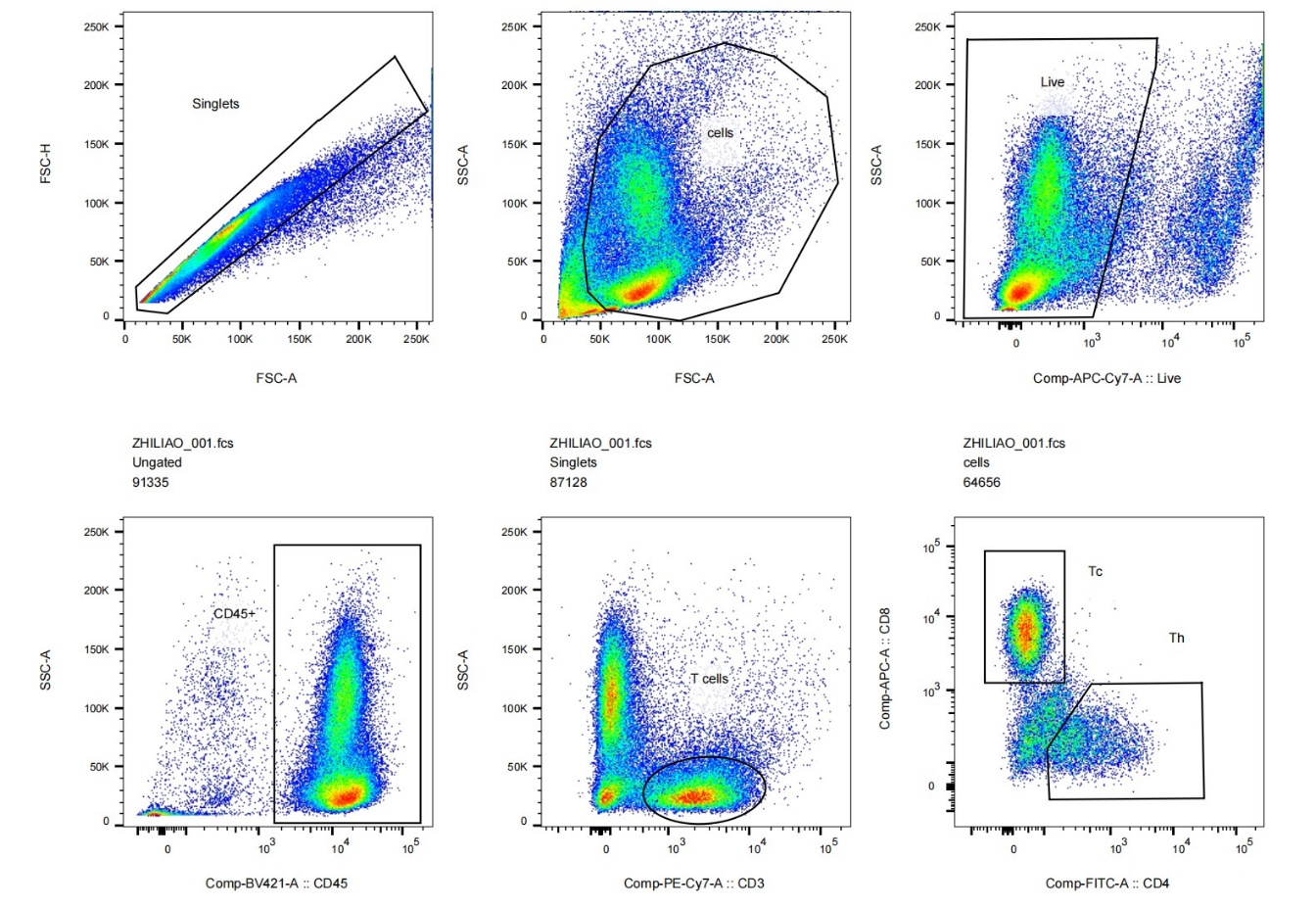


Figure. S11. Flow cytometry analysis of tumor tissue and loop strategy flow chart. The figures show the process of gradually screening cells after flow cytometry triple staining.


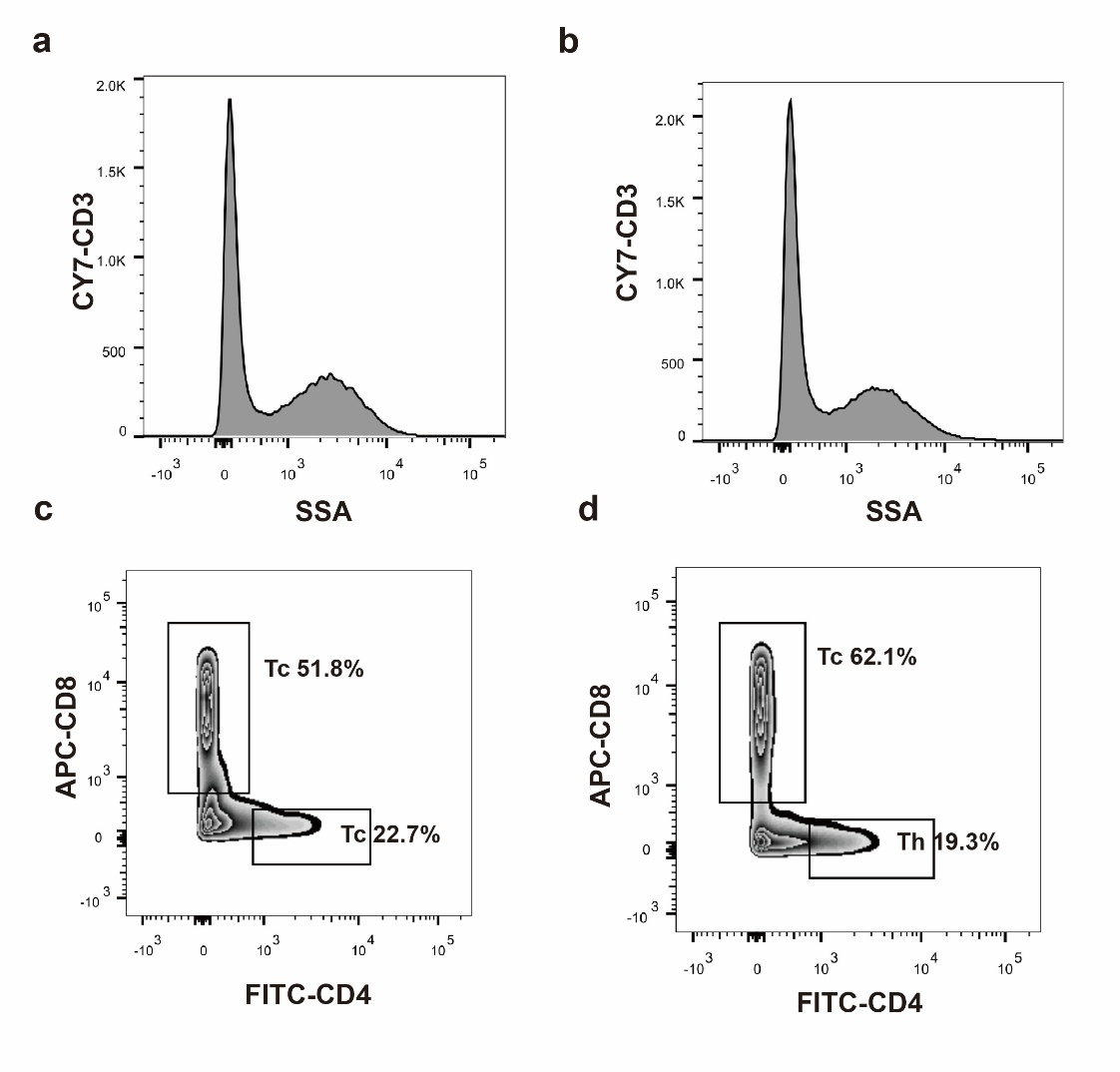


**Figure S12.** The changes of T cells and their subsets in HCC tissues after Pc@Zr-MOF mediated sonodynamic therapy were analyzed by flow cytometry. a, b) T cell histograms were analyzed by flow cytometry before and after therapy. c, d) Zebra map of Tc and Th cell distribution by flow cytometry.


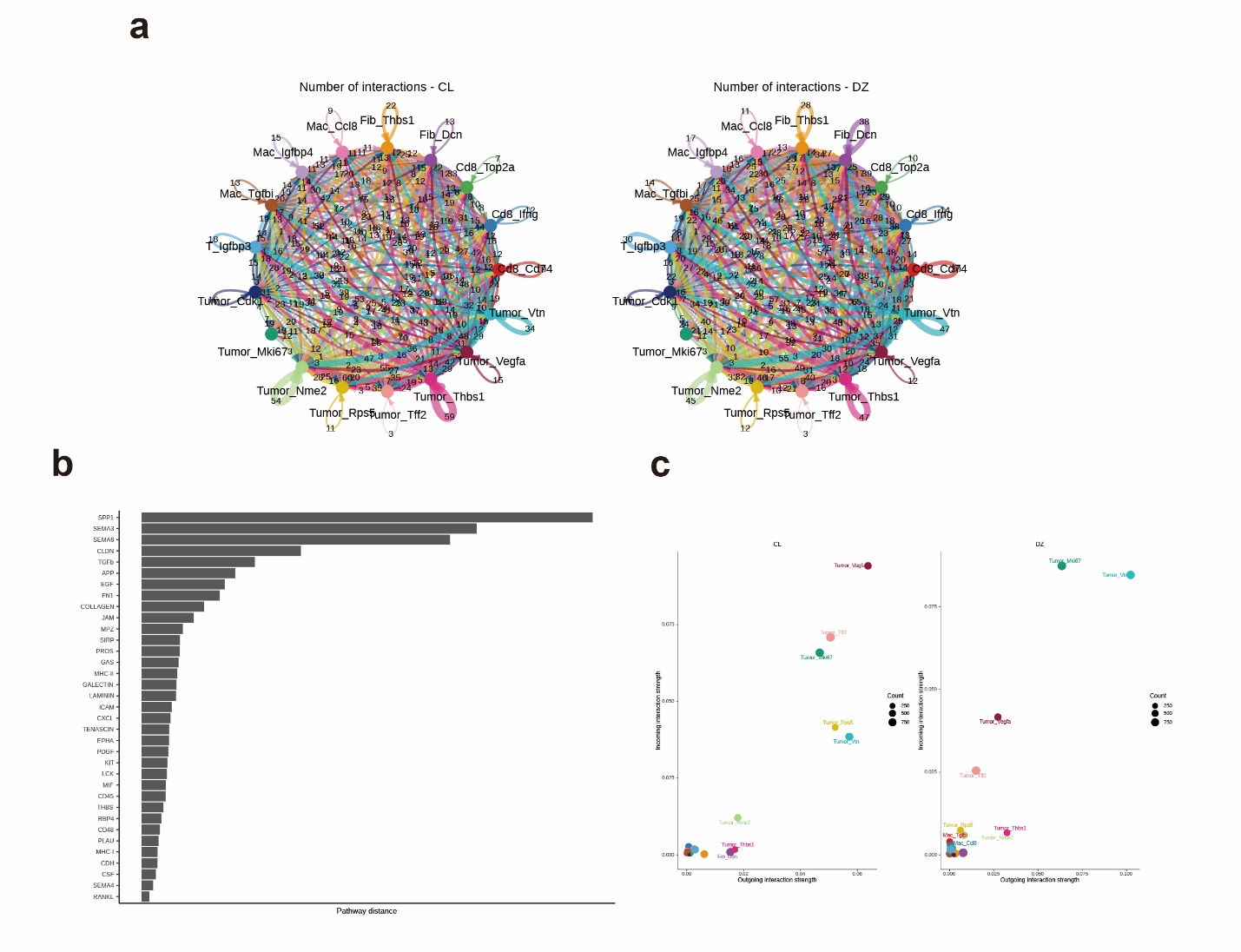


**Figure S13.** Cell interaction analysis and overall enrichment of cell communication signals. a) Cell interaction communication analysis spherical map. b) Bar graph of cell communication enrichment of overall cells. c) Map of incoming and outgoing interaction between treatment and control groups.
